# Correction: Resilience, ingenuity, and identity: A multi-level analysis of the Filipino community health worker experience in rural and remote municipalities in the Philippines

**DOI:** 10.1371/journal.pgph.0005699

**Published:** 2025-12-22

**Authors:** Regine Ynez H. De Mesa, Zoé Mistrale Hendrickson, Carol Stephanie C. Tan-Lim, Anton Elepaño, Noleen Marie C. Fabian, Johanna Faye E. Lopez, Carl A. Latkin, Leonila F. Dans, Mia P. Rey, Antonio Miguel L. Dans

The first author, Regine Ynez H. De Mesa, is incorrectly noted as the corresponding author. The correct corresponding author is Zoé Mistrale Hendrickson. Zoé Mistrale Hendrickson’s email address is: zhendri1@jhu.edu.
