# Supplementary material for: Resilience, ingenuity, and identity: A multi-level analysis of the Filipino community health worker experience in rural and remote municipalities in the Philippines
Source: PLOS Glob Public Health. 2025 Aug 18;5(8):e0004965. doi: 10.1371/journal.pgph.0004965 (PMC12360505; doi:10.1371/journal.pgph.0004965)
Supplement: S1 Appendix — (DOCX) [file pgph.0004965.s001.docx]

**Voices from the Field: Understanding the Lived Experiences and Capacity-Building Needs of Community Health Workers in Rural and Remote Philippines**

National Academy of Science and Technology Protocol Proposal

Developed by Regine Hughes De Mesa

**1.0 INTRODUCTION**

**1.1 Abstract**

This protocol proposal outlines a study aimed at investigating the lived roles of community health workers (CHWs) in delivering primary care services in rural and remote areas of the Philippines, exploring their capacity-building needs, and examining their perceptions of using UpToDate as a tool to support their work. The study will be conducted using a qualitative research design, combining focus group discussions and key informant interviews among CHWs in a rural and remote municipality in the Philippines. A target sample of 24 CHWs will be purposively recruited based on their tenure as CHWs and prior exposure to UpToDate capacity-building interventions. The data will be analyzed iteratively using a multi-stage approach outlined by Braun and Clarke (2006) to identify the themes embedded within the data set. The study's findings may inform future interventions to improve CHWs' effectiveness in delivering primary care services in underserved areas in the Philippines.

**1.2 Study Objectives**

The objectives of this study are to: 1) outline the roles that rural and remote community health workers (CHWs) occupy in delivering primary care services in the Philippines; 2) identify the capacity-building needs of CHWs for their roles; and 3) explore CHWs' perceptions of using UpToDate as a capacity-building tool.

**2.0 METHODOLOGY**

**2.1 Study Setting**

The proposed study will serve as an extension of an existing collaboration between the Philippine Primary Care Studies (PPCS) program and the municipalities of Samal in Bataan, and Bulusan in Sorsogon. PPCS is a collection of studies designed to enhance primary care services in various settings throughout the country. To streamline participant recruitment and leverage contextual data obtained from previous communication, the proposed study will be conducted among the network of CHWs at the rural and remote PPCS pilot sites.

This study will investigate the experiences of CHWs employed in publicly-funded primary care systems in two municipalities in the Philippines. In a study led by PPCS in 2019, it was found that there were 100 CHWs employed by municipalities in the rural pilot site, and 126 in the remote pilot site (Bernal-Sundiang et al., 2022). These two pilot sites comprise 14 and 24 smaller barangay units, respectively, to which CHWs support primary care services. In these settings, CHWs provide frontline support to patients in accessing care and are similarly tasked to facilitate a range of public health initiatives (e.g. patient education, mobilizing immunization programs, and in-facility support).

**2.2 Study Design and Instrumentation**

This study aims to explore the intersection between the lived experiences of CHWs and their unmet capacity-building needs while performing their duties. To achieve this, the research will employ a qualitative research design using a combination of focus group discussions (FGDs) and key informant interviews (KIIs). The FGDs and KIIs will explore the motivations of CHWs in delivering primary care services and identify strategies to better support them in their work. A semi-structured interview guide, based on existing literature, will be adapted to suit the local contexts of rural and remote municipalities in the Philippines. Furthermore, this guide will be tailored to the experiences and language of CHWs in these areas (see Appendix A).

The FGDs and KIIs will be conducted in the lingua franca spoken by the participants in each study site, namely Filipino (Tagalog) in Bataan and Bikolano in Sorsogon. Each session will be led by a trained member of the research team and will last between 60 to 90 minutes. Although FGDs will be the primary method of data collection, KIIs will be utilized when FGDs are not feasible.

**2.3 Proposed Data Analysis**

Data collection and analysis will be conducted iteratively to continuously refine the research questions and identify emerging themes. A medical anthropologist and a research-trained member of the community will be present in overseeing data collection for the planned FGDs and KIIs. Moreover, the investigators aim to involve a CHW from each site as a member of the steering committee for this study. The data collection methods and analysis will be co-produced with both technical and lay teams, ensuring that the perspectives and expertise of all stakeholders are incorporated into the research process. Data collection for this study will be undertaken through in-person or online FGDs/KIIs depending on the availability of each participant.

The FGDs and KIIs will be audio-recorded with the consent of all participants. A clean verbatim transcription of FGDs and KIIs will be produced following each session and subsequently uploaded to MAXQDA for analysis. These extracts, potentially recorded in a mix of English, Filipino (Tagalog), and Bikolano, will then be contextually translated to English in their entirety. Following the methods outlined by Braun and Clarke (2006), the investigators of this study will pursue a multi-stage approach in analyzing convergent and divergent themes present in the data set. Figure 1 provides an overview of the planned analysis for this study.


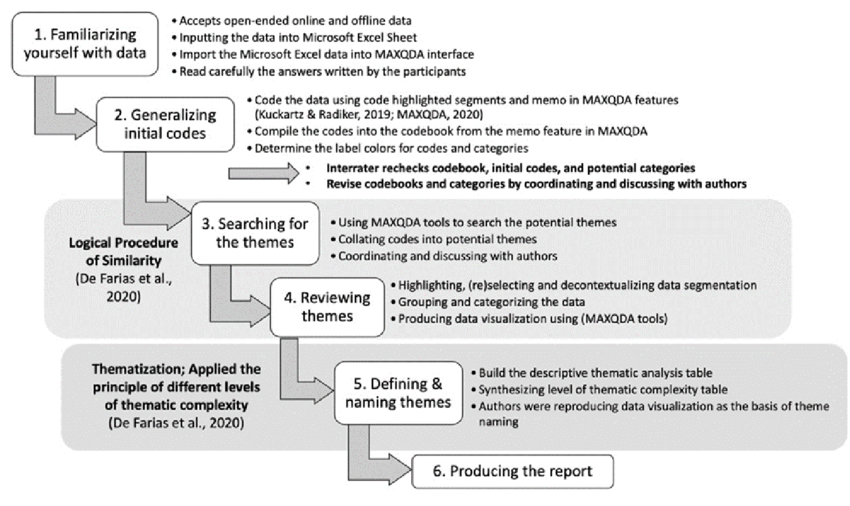


Fig. 1 Thematic analysis method outlined by Braun & Clarke (2006)

The lead researcher will read and openly code all FGD and KII transcripts. As a check for validity, 50% of the transcripts will be double-coded by a second researcher on the team. This tandem of researchers will be subsequently referred to as the coding team. Once initial codes have been derived from the data set, the preliminary set of unified codes will be jointly discussed by the coding team and modified as needed. The coding team and CHW partners will then collaboratively work towards extracting the salient themes from the identified codes. These themes will be reviewed, defined, and presented to the broader research team for feedback before manuscript development.

2.4 Sampling

A minimum target sample of 12 CHWs per site or 24 CHWs in total will be recruited for the planned study. To ensure a diverse range of experiences is represented in the dataset, participants will be recruited purposively based on their tenure as a CHW within the municipality. Table 1 provides an overview of the sampling frame proposed for this study. It is critical to note that classifications of short, medium, and long tenure are subjectively provided and only serves as a guide for recruitment. While researchers will remain cognizant of ensuring the participant pool remains as diverse as possible, this sampling frame will be adapted depending on the roster of CHWs present in the workforce.

Table 1. Sampling frame for recruiting CHWs for FGD/KII participation

| **Length of Tenure** | **Rural Site** | **Remote Site** | **Subtotal** |
| --- | --- | --- | --- |
| Short tenure (<3 years) | 4 | 4 | 8 |
| Medium tenure (3-6 years) | 4 | 4 | 8 |
| Long tenure (>6 years) | 4 | 4 | 8 |
| **Total** | **12** | **12** | **24** |

Table 2 presents a summary of inclusion and exclusion criteria for participant recruitment. As one of the three objectives of this study is to elicit participants' perceptions on more recent PPCS interventions, namely UpToDate access and journal clubs, all participants must have had prior exposure to UpToDate. This exposure can take the form of independently accessing UpToDate or attending any of the bi-monthly journal clubs, interventions which were present in these two communities from 2021 to present.

Table 2. Inclusion and exclusion criteria for participant recruitment

| **Inclusion Criteria** | **Exclusion Criteria** |
| --- | --- |
| - CHWs currently employed under the municipality of Samal, Bataan and Bulusan, Sorsogon. - Consenting CHWs (18 years old and above) in each of the study sites. - CHWs who had prior exposure to UpToDate, either in the form of accessing UpToDate or attending bi-monthly journal clubs. | - Non-consenting CHWs or individuals unable to freely provide informed consent. - CHWs with physical or mental dispositions that inhibit participation in in-person or online FGDs or KIIs. - Former CHWs who have retired before the data collection period begins. - CHWs who are not actively facilitating primary care services or providing primary care support. |

**3.0 Ethical Considerations**

3.1 Ethics Clearance

This study will adhere to the National Ethical Guidelines for Health and Health-related Research of 2017, as well as the Good Clinical Practice Guidelines. As the proposed study serves as an extension of the original PPCS research objectives, it will be covered by the PPCS ethics clearance under the University of the Philippines Manila Research Ethics Board (UPMREB). The necessary correspondences to UPMREB will be initiated by the principal investigator assigned for this study and under PPCS. The principal and co-investigators of this study have no relevant conflicts of interest to declare.

3.2 Consent, Participant Benefits, and Participant Risks

Trained FGD/KII facilitators will elicit written consent from all CHWs before commencing data collection. To ensure that the consent is voluntary, the facilitators will also verbally explain the purpose of the study and the intended use of the data obtained in a language and register understandable by lay audiences. Additionally, the facilitators will allow for a brief period during which potential participants may ask questions.

Since the total duration of the intended FGDs/KIIs can range between 60-90 minutes, CHWs will be provided a small financial incentive for their time. The investigators foresee no added risk to participants that can result from participating in the study. To prevent COVID-19 transmission, all FGDs and KIIs will be conducted in well-ventilated and preferably outdoor spaces. Participants or researchers who exhibit flu-like symptoms immediately before or during the FGDs/KIIs will not be allowed to continue participation.

3.3 Withdrawal Criteria

This study operates on the principle of voluntary participation. As such, participants have the right to withdraw at any point during the study without penalty or consequence. Additionally, the researchers retain the right to terminate the study at any time, should the need arise.

3.4 Data Management and Privacy

The research team, including research partners, will be subject to a written non-disclosure agreement prior to study engagement. Furthermore, researchers involved in handling raw data (i.e. audio recordings and transcripts) will receive comprehensive training on data privacy and management. Upon transcription, all personally identifiable information (PIIs) will be replaced by unique research identification codes. While a master list with PIIs will be maintained to back-up anonymized data, access to this master list will only be made available to the coding team. Electronic research data will be stored in a limited access OneDrive folder – this includes all audio recordings, field notes, analyses, and final research reports generated from the study. In the event physical study data is available, these documents will be securely stored in a locked room in the University of the Philippines Center of Integrative Development Studies building. Contact lists and participant recruitment records will be destroyed after the research manuscript has been finalized by the study team.

**4.0 Timeline**

This study targets a 7-month timeline from inception to manuscript development. This timeline covers the period for institutional review, preparation, data collection, analysis, and manuscript development.

Table 8. Proposed activities and target timeline of events

| **Activities** | **0** | **1** | **2** | **3** | **4** | **5** | **6** | **7** |
| --- | --- | --- | --- | --- | --- | --- | --- | --- |
| Institutional review |  |  |  |  |  |  |  |  |
| Preliminary study preparations |  |  |  |  |  |  |  |  |
| Hiring and training research assistants |  |  |  |  |  |  |  |  |
| Recruitment and facilitation of FGDs and KIIs |  |  |  |  |  |  |  |  |
| Data transcription and translation |  |  |  |  |  |  |  |  |
| Data analysis and processing |  |  |  |  |  |  |  |  |
| Manuscript development |  |  |  |  |  |  |  |  |

**Appendix A**

Semi-Structured Topic Guide for Focus Group Discussions and Key Informant Interviews

Adapted from Mallari et al (2020) and Rodriguez et al (2022)

**I. Clarifying Roles of Community Health Workers** (*Objective 1)*

1. How long have you been working as a Barangay Health Worker (BHW) in this municipality?
2. What motivated you to volunteer as a BHW?
3. What are your daily responsibilities and activities as a BHW?
4. Who is responsible for supervising your work and providing guidance on your tasks?

**II. Identifying Capacity Building Needs: Barriers & Enablers in Role Performance** *(Objective 2)*

1. What do you find most rewarding about your job?
2. Do you feel adequately recognized and compensated for your work?
3. If you could change any of your current responsibilities, what would you change?
4. What is the most significant challenge you face in your job?
5. What resources do you believe would better support you in fulfilling your responsibilities?

**III. Perceptions on UpToDate as a Tool for Capacity Building** *(Objective 3)*

1. Do you have ongoing opportunities for training or continuing professional development?
2. Have you used UpToDate outside trainings or journal clubs?
   1. What motivated you to access UpToDate on your own?
   2. Alternatively, what preventing you from accessing UpToDate on your own?
3. What advantages have you experienced from using UpToDate and attending journal clubs?
4. If given the chance, what changes would you make to UpToDate or the journal clubs?

**IV. Closing**

1. Is there anything else you wish to share about your role, the challenges you encounter at work, and your views on UpToDate and the journal clubs?
2. Can we contact you in the future if we have follow-up questions?
