# Supplementary material for: Resilience, ingenuity, and identity: A multi-level analysis of the Filipino community health worker experience in rural and remote municipalities in the Philippines
Source: PLOS Glob Public Health. 2025 Aug 18;5(8):e0004965. doi: 10.1371/journal.pgph.0004965 (PMC12360505; doi:10.1371/journal.pgph.0004965)
Supplement: S1 File — (ZIP) [file pgph.0004965.s002.zip › 2023-07-09 PPCS GIDA Translation FGD 2.docx]

**Focus Group Discussion Transcription**

FGD 2 – Medium Tenure

**Philippine Primary Care Studies**

NAST CHW Experience Study

**PRELIMINARY INFORMATION**

| Location: | Rural Health Unit of the Remote site located in Southern Luzon |
| --- | --- |
| Date Recorded: | July 9, 2023 |
| Transcriber’s Remarks: | The informant’s name and key identifiers have been redacted from this transcription. |
| List of Acronyms: | NDP = Nurse Deployment Program  BHW = Barangay Health Worker  UTD = UpToDate  BNS = Barangay Nutrition Scholar  HRH = Human Resources for health  BHC = Barangay Health Center |

**TRANSCRIPTION**

**--[Begin Transcript (00:01:48)]—**

| ***IN:*** | *We have work from Monday-Friday right? Can you share your daily routine during weekdays as Barangay Health Worker?* | | |
| --- | --- | --- | --- |
|  | **Daria:** | We have a schedule for every duty. What I usually do is BP monitoring, getting IPR, or handling accidents such as minor wounds. |  |
| ***IN:*** | *So that’s your usual task for the whole day?* | | |
|  | **Daria:** | Yes, Ma’am. |  |
| ***IN:*** | *When not on duty, what do you usually do?* | | |
|  | **Daria:** | When there is a meeting in the barangay, I always attend. This week, I am facilitating the provision of [a government-issued document]. |  |
| ***IN:*** | *Alright, Ma’am. Who else wants to share? How about you, Ma’am Bella? What do you usually do?* | | |
|  | **Bella:** | During my duty, I facilitate BP Monitoring and weighing of patients. Or if there is no patient, I clean the premises of the center or wash the dishes. |  |
| ***IN:*** | *So, your tasks are all around? Aside from seeing patients, you also do chores inside the center.* | | |
|  | **Bella:** | Yes, Ma’am. |  |
| ***IN:*** | *But it is not done daily?* | | |
|  | **Bella:** | Yes, we have a monthly schedule, especially for the monitoring of children. |  |
| ***IN:*** | *Alright, how about you, Ma’am Cess, what are your responsibilities?* | | |
|  | **Cess:** | When there are important tasks to do…[inaudible] |  |
| ***IN:*** | *When you are in the barangay, what do you usually do daily? What are your tasks?* | | |
|  | **Cess:** | Conduct BP Monitoring as needed or refer patients to RHU. I also attend to my children. |  |
| ***IN:*** | *Oh, so you bring your child to work? How about you, Ma’am Fiya?* | | |
|  | **Fiya:** | The first thing I do is clean the center and facilitate BP monitoring if there’s a patient who needs it. I also assist visitors in the center or barangay. |  |
|  | **Daria:** | Or anything related to health…we also assist visitors in house-to-house visits. |  |
| ***IN:*** | *This is because you are the ones who know the houses and people, right?* | |  |
|  | **Fiya:** | Yes, if you are looking for anyone, BHW knows well where to find them. |  |
| ***IN:*** | *Okay, how about you Ma’am Ariel?* | |  |
|  | **Ariel:** | When I arrive at the center, I clean the premises as early as 7 am before patients arrive at 8 am. I facilitate BP monitoring; however, I am usually alone in the center. I sometimes visit houses for BP Monitoring, especially the senior citizens who can no longer afford to leave the house. We also help each other during [a locally initiated immunization program]. |  |
| ***IN*** | *Is the house-to-house visit not every day? Or do you only visit as needed especially for senior citizens and PWDs?* | |  |
|  | **Ariel:** | Yes, Ma’am. I have one patient who really is hardheaded. He lives alone and no one looks after him so we are the ones checking on his welfare, however, we’re having a hard time managing him since is non-adherent to our advice. |  |
| ***IN*** | *It really takes a lot of patience, right?* | |  |
|  | **Ariel** | Yes, there was a time when he got into an accident in the house causing a concussion on his head. Residents in the barangay got curious as to why the patient hasn’t been out for days so they reported it to the barangay captain to have him checked. That is when we learned that he was unconscious due to a head injury. |  |
| ***IN*** | *Oh, no. That was dangerous. He should not be left alone* | |  |
|  | **Ariel:** | Yes, Ma’am, however, no one lasts since he is a very problematic person. |  |
| ***IN*** | *Yes, Ma’am. There really are patients who are non-adherent, especially the senior citizens and PWDs. Those are the usual challenges in the barangay. How about you, Ma’am Ella?* | |  |
|  | **Ella** | My duty is in [specific health center assignment] every Wednesday. When there is an activity like [locally initiated immunization program], or child deworming, we are always present to assist. My tasks include BP monitoring on the elderly, referring patients, and facilitating telemedicine which is very helpful especially if there are many patients seeking consultation.  This July, we are busy preparing for nutrition month wherein there are a lot of activities in line that we need to participate in. Our work goes beyond as BHW, whatever the community needs, we assist. I also do rove of patients in the morning for monitoring. I have 54 households to monitor in Purok 4. The other day, I visited a patient with postpartum and referred her to RHU for additional intervention. I also attend [community event] as needed  We don’t just focus on health, but rather, we extend as we can to help others especially during emergencies. |  |
| ***IN*** | *Thank you, Ma’am. That is true. As a healthcare worker, you are serving the front lines, and you play a huge part in the community. That’s what [organization] is trying to highlight. This is also why we keep on pushing to give you training since you are needed In the community.* | |  |
|  | **Daria** | Yes, we are the first point of access for the patients, especially for first-aid treatment. |  |
| ***IN*** | *Yes, Ma’am.* | |  |
|  | **Fiya** | There are also hard-headed [infectious disease program] patients, it seems like they do not want to get healed. |  |
|  | **Ariel** | Same with my [infectious disease program] patient, he keeps on smoking and drinking. |  |
| ***IN*** | *There really are non-adherent patients. That is already their vices or addiction.* | |  |
|  | **Ariel** | Yes, but it isn’t good for the family members in the household. |  |
| ***IN*** | *Yes, Ma’am. Secondhand smoke is equally dangerous or even worse.* | |  |
|  | **Fiya** | In my case, I usually encounter pregnant women that are about to labor or give birth. Even in the wee hours of the morning, there is a call of duty. |  |
| ***IN*** | *When you are a BHW, you are always on call. You should be ready any time since emergencies choose no time.* | |  |
|  | **Bella** | That’s true. Even when you are in the middle of house chores or late at night, you will be called. |  |
|  | **Ella** | Especially for those who are about to give birth. So even if it is late at night, or there is a dog, nothing will stop you. |  |
|  | **Daria** | Same with me. I just got used to it. |  |
|  | **Ariel** | Yes, Ma’am. Even with no undergarments on or if your clothes are torn, you will have to immediately leave and assist the patient. |  |
|  | **Fiya** | There was also a time when I had to accompany a patient to the provincial office in [another city]. |  |
| ***IN*** | *There are really a lot of sacrifices to make.* | |  |
|  | **Ariel** | Yes, especially if there are accidents. The BHWs are the first point of access. |  |
|  | **Fiya** | I experienced accompanying a patient before for two days. I have no spare clothes or underwear. The patient doesn’t want me to leave her. That’s why now, I already ensure to always keep extra clothes in my bag just in case so I am prepared. |  |
|  | **Ariel** | In my case, tho it really is hard, I find it fulfilling to be able to help other people. |  |
| ***IN*** | *The common emergency you encounter is giving birth, right? So some of you here have already been serving for years, what motivates you to stay despite these challenges?* | |  |
|  | **Daria** | So, we can help more people. It feels good. |  |
|  | **Ariel** | I can share my knowledge with others. |  |
|  | **Ella** | Also, when you love your job and you are happy with it, there’s nothing impossible. |  |
| ***IN*** | *That’s true, Ma’am. If you really like your job, you can do anything no matter how hard it is.* | |  |
|  | **Fiya** | Like right now, we are happy to be together. |  |
|  | **Daria** | Before, when I wasn’t yet a BHW, I was pedantic, but now that I am already in the service, I am learning a lot. |  |
| ***IN*** | *Would you say that being a BHW made you more fulfilled in work?* | |  |
|  | **Daria** | Yes, and it also gives me a sense of trust and self-confidence in engaging with other people. |  |
| ***IN*** | *Yes, especially as a BHW, you will have to entertain different kinds of people. How did you become a BHW?* | |  |
|  | **Ariel** | In my case, I was just recruited. It just happened that one BHW left and so they needed a replacement. I agreed to be one so I can just try it and then eventually I started to love it. |  |
| ***IN*** | *So it was your choice and not because it was enforced on you?* | |  |
|  | **Ariel** | Yes, Ma’am. It was my choice to agree to be recruited since someone has to replace me. |  |
| ***IN*** | *Then you eventually liked it since you lasted for years? How about you, Ma’am Cess?* | |  |
|  | **Cess** | I was also recruited since a lot of BHWs were removed before. I agreed because I’m also initially after the salary and experience, and also it is an opportunity for me to help and serve others aside from it is also an avenue for self-development. |  |
| ***IN*** | *I see. So, you just learned to love it eventually.* | |  |
|  | **Cess** | Yes, Ma’am. It’s about serving others even tho we have to go so far just to visit their houses. |  |
|  |  |  |  |
| ***IN*** | *How about you, Ma’am Bella?* | |  |
|  | **Bella** | In my case, I was just recruited by my BNS. |  |
| ***IN*** | *Was it your choice to agree or be recruited?* | |  |
|  | **Bella** | It was offered to me. I wanted to try it as well and then I eventually enjoyed it, especially the house-to-house visits, attendance to seminars/training, and the additional learning that comes from it. It is also fun to interact with fellow BHWs even though our work requires us to stay in quarantine areas for days. |  |
| ***IN*** | *Aside from those you have mentioned, what else do you think are the benefits of being a BHW?* | |  |
|  | **Ella** | We receive an incentive every December and during seminars that we attend; we are entitled to an allowance as long as we have a certificate of appearance as proof of attendance. |  |
| ***IN*** | *Oh so you can claim the travel expenses but subject to the availability of funds in the barangay* | |  |
|  | **Bella** | In my case, I don’t think about those. I’m just after the training. |  |
| ***IN*** | *The training itself is the incentive already along with the learning and insights you’ll gain. How about the others?* | |  |
|  | **Ella** | We are provided with supplies of vitamins. |  |
|  | **Ariel** | We also receive relief goods every year. That is one sack of rice and 300 php cash from the provincial government. |  |
| ***IN*** | *At least there is a reward for your sacrifices.* | |  |
|  | **Daria** | I really do hope as well that the magna carta for BHW be passed as well as this means more benefits for us. |  |
| ***IN*** | *I really hope so too. It will be really beneficial for BHWs.* | |  |
|  | **Daria** | Yes, Ma’am. Our travel allowance and incentives will increase too.  . |  |
|  | **Ariel** | Yes, although passing that bill or law will still take more time. |  |
|  | **Daria** | I hope that the promised incentive worth PHP 1,500.00 from the provincial government will be realized this December. |  |
| ***IN*** | *Incentives and training are indeed good motivations. It is also beneficial, especially for sustaining the daily needs of your family. Now that you’ve mentioned the benefits of being a BHW, do you think that the kind of working environment you have affected that? Who are your supervisors?* | |  |
|  | **Ella** | The midwife or nurse. |  |
| ***IN*** | *Do you think that having a good working relationship matters?* | |  |
|  | **Ella** | For me, that is the most important one. You need to be comfortable with your colleagues so you can work efficiently, although, there are times that you won’t really get along with some of them due to differences but until you can handle them, you just go do your job. I just pray that I will be guided always to handle such things because it really happens, with so many tasks at hand. For me, you just do your job and work hard because if you do, you will find a sense of fulfillment as a BHW. As long as you are happy with your accomplishments and training, that is something that is priceless since you worked hard for it, especially the effort I make in attending the UTD Journal clubs every Thursday in which I learned so many things. |  |
|  | **Cess** | Yes, because the topics are the usual cases we encounter. |  |
| ***IN*** | *Yes, those are really beneficial in your work as BHWs.* | |  |
|  | **Daria** | Yes, Ma’am. These are additional learning and different approaches to handling patients. |  |
| ***IN*** | *It’s like UTD serves as your bible or dictionary whenever you need a reference.* | |  |
|  | **Ella** | Yes, it gives you the idea to properly manage patients. |  |
| ***IN*** | *Yes, Ma’am. You are able to properly explain to patients these cases, and show them that you are credible thus earning their trust. How about you, Ma’am Ariel?* | |  |
|  | **Ariel** | We don’t have any issues in our working relationship so far. |  |
| ***IN*** | *Do you think having a good working environment is beneficial to you?* | |  |
|  | **Ariel** | Yes, it is. I have a good relationship with my supervisor and she sometimes told me not to overwork. |  |
| ***IN*** | *It’s good that you are already comfortable. How about you, Ma’am Fiya?* | |  |
|  | **Fiya** | *It’s fun although problems arise sometimes such as not getting along with other BHWs but that is normal.* |  |
| ***IN*** | *How do you handle that?* | |  |
|  | **Fiya** | I just do my job. |  |
| ***IN*** | *Working environment really plays a huge part. It’s good to know that you are comfortable in your working place, how about you, Ma’am?* | |  |
|  | **Daria** | Same with her since we belong to the same barangay. We do have a good working relationship. |  |
| ***IN*** | *Are the BHWs in your area been in the service for years already?* | |  |
|  | **Fiya** | Yes, except for the new one. |  |
| ***IN*** | *How about you, Ma’am Bella?* | |  |
|  | **Bella** | Same with me, Ma’am. |  |
| ***IN*** | *How about you, Ma’am Cess? How was your experience so far having been in the service for 3 years?* | |  |
|  | **Cess** | is Just fine. There are some who lack motivation sometimes and there are some who you won’t get along with. |  |
| ***IN*** | *Okay, is there anything else you want to add relating to your work environment? If none, let’s proceed to the next question. What resources do you believe would better support you in fulfilling your responsibilities?* | |  |
|  | **Daria** | We need a laptop and a cellphone. |  |
| ***IN*** | *That’s correct, Ma’am. Especially since we are using UTD and attending journal clubs. Everything is also mostly online these days.* | |  |
|  | **Ella** | Yes, laptops are very important since everything is electronic already. |  |
| ***IN*** | *But you do have a laptop in the barangay?* | |  |
|  | **Daria** | Only in the barangay hall but not in the health center. |  |
|  | **Ella** | Also, we are unable to use or borrow it frequently. |  |
|  | **Bella** | Only the BNS has a laptop since they need it for the monthly reports for submission. |  |
|  | **Ella** | We need it, especially for seminars or workshops. |  |
| ***IN*** | *That’s true, especially, since the mode of communication these days is mostly online or via social media platforms. However, another challenge is the problems with the signal. Other than that, are there more?* | |  |
|  | **Ariel** | Medicine supply and equipment, Ma’am. Hopefully, we can be provided with our own BP apparatus so we can use it during house-to-house visits since the ones in the center are being used by walk-in patients too. |  |
|  | **Bella** | Pulse oximeter too. |  |
|  | **Fiya** | Like the other night, there is an emergency and the patient’s BP must be checked. We still have to go to the center and ask for the keys from someone, just to get it. |  |
|  | **Ella** | There should be one designated for the center and one for each BHW. There are also cases where the apparatus is missing or broken. This is why we have logbooks for those using it. |  |
|  | **Ariel** | For example, the nebulizer is missing. We refer to the logbook to track who is the one who borrowed it last. |  |
| ***IN*** | *At least you have a log book to refer to and trace those who borrowed it. Other than those, are there any issues or problems that you encountered?* | |  |
|  | **Daria** | Whenever there is a distribution of relief goods, some people get mad if they are not included in the list without understanding the reasons for it. |  |
|  | **Ariel** | Just to add on that too, BNS and BHWs also have separate assistance. BNS usually receive earlier than BHWs. |  |
| ***IN*** | *Does this create a gap between the two parties?* | |  |
|  | **Ariel** | Yes, Ma’am. It should not be like that since we only work in one workplace. BHWs and BNS work together in accomplishing the reports. |  |
|  | **Ella** | We should be equal as we receive the same amount of salary. |  |
| ***IN*** | *Aside from what you have mentioned, are there any challenges you would like to add? So far, you’ve mentioned the scarcity of supply, engagement with patients, etc.* | |  |
|  | **Ella** | Perhaps, one is managing the [infectious disease program] Patients. No matter how you guide them, there are still some who are hard-headed and would continue smoking without realizing that if you do not take yourself seriously, things can get worse. However, we have to be lenient as well. I usually use a reward system to convince them to follow. |  |
| ***IN*** | *It really takes a lot of convincing.* | |  |
|  | **Ariel** | It’s frustrating that no matter what sacrifices you make, some patients don’t care and won’t even bother to take care of themselves. |  |
|  | **Bella** | You really need to be patient. |  |
|  | **Daria** | Especially when patients take medications and their bodies start to feel the effects, this deters them from continuing it since they feel like it makes them weak instead. |  |
| ***IN*** | *I see. They don’t know that side effects are normal so you really have to make them understand.* | |  |
|  | **Ella** | It’s really expected, especially if it is the first time. You really have to make them understand and educate them about the side effects. |  |
| ***IN*** | *So, these are the usual problems you encounter, making the patient understand and convincing them. Now, If you could change any of your current responsibilities, what would you change?* | |  |
|  | **Ella** | None perhaps but more on additional training. |  |
|  | **Daria** | I agree, Ma’am. Just add more. |  |
|  | **Ariel** | Like that one convention promised to us that should have been conducted last April. |  |
| ***IN*** | *Is this planned by the provincial government?* | |  |
|  | **All** | Yes. |  |
| ***IN*** | *Everyone feels satisfied and won’t change anything. Alright.* | |  |
|  | **All** | Yes, Ma’am. Just add more. |  |
|  | **Ella** | We also hope doc dans can come to visit us again for f2f training. |  |
| ***IN*** | *We also hope so, Ma’am, but How do you feel about the use of technology or medical software for your scope of practice?* | |  |
|  | **ELLA** | It is useful even with no patients since you can do advance readings so that when a patient comes in, you already have an idea and you can easily refer to them. UTD journal club is beneficial and it also strengthens our relationship. |  |
| ***IN*** | *Are you comfortable using these applications? Same with UTD and EMR?* | |  |
|  | **Ella** | Yes, since it is convenient when it is online or if the data is in sync. You can already see the data of the patient once referred to RHU. |  |
|  | **Ariel** | The data is already synced wherever the patient goes. |  |
| ***IN*** | *Same with the referral system that primary care has been initiating, whether it is in the pharmacy or laboratory, everything is already systematic. For instance, in a pharmacy, the pharmacist can only dispense the medicines prescribed by the physician.* | |  |
|  | **Ella** | You can easily submit the data and see the history of the patient. |  |
|  | **Daria** | The only problem is the signal. |  |
| ***IN*** | *Yes, so until the connection or internet is resolved, we cannot fully utilize it.* | |  |
|  | **Ella** | Yes, Ma’am. Telemedicine is also useful and easy to refer patients |  |
| ***IN*** | *Yes, at least in telemedicine, you can just call or text. We are already done with sharing your roles as BHW, is there anything else you want to share before we proceed to the next?* | |  |
|  | **All** | None so far. |  |
| ***IN*** | *Okay, this time, let’s talk about your perception of using UTD. How often do you use it?* | |  |
|  | **Daria** | I just use other people’s devices to access it. |  |
| ***IN*** | *Did everyone attend the journal club?* | |  |
|  | **ALL** | Yes, Ma’am. |  |
|  | **Daria** | We regularly attend since we have a duty every Thursday. |  |
| ***IN*** | *How often do you use it tho? When did you last use it?* | |  |
|  | **Bella** | Last year |  |
|  | **Cess** | Just once. |  |
| ***IN*** | *Were you able to use it in handling or managing patients?* | |  |
|  | **Ariel** | Yes, because we use it as a reference and reading material. |  |
|  | **Daria** | If there is a patient, I usually use UTD as a reference using the cellphone provided by primary care since the app is already installed there. |  |
| ***IN*** | *Do you regularly attend UTD?* | |  |
|  | **All** | Yes. |  |
|  | **Fiya** | We even have certificates although we are yet to print it. |  |
|  | **Ariel** | Sometimes, we are unable to attend due to being busy. |  |
|  | **Cess** | We were able to attend the training in [another province] before. |  |
| ***IN*** | *How was the experience?* | |  |
|  | **Daria** | It is helpful, especially the topics on health cases such as sore eyes. |  |
|  | **Bella** | It is nice since we learn a lot from it. |  |
|  | **Ella** | Then we were also to interact with the BHWs [throughout the municipality]. |  |
| ***IN*** | *If given the chance, what changes would you make to UpToDate or the journal clubs? What else do you think can be improved? What could be the reason why some of you are unable to use it?* | |  |
|  | **Ariel** | My cellphone is not compatible with UTD. |  |
|  | **Fiya** | We usually use the donated phone of Primary Care, however, it doesn’t always have a load. |  |
|  | **Ariel** | Ours is connected to the wifi that’s why we can use it. |  |
| ***IN*** | *Okay, it seems like everyone does not have any changes they wish to make for UTD. Let’s proceed with the next question. What training opportunities for training or continuing professional development are available to you?* | |  |
|  | **Ella** | It would be better if it is face-to-face training so we can understand the information more effectively. That’s why we appreciate you visiting us and teaching us, especially in facilitating telemedicine which has been very helpful during the time when there was a hand and foot disease outbreak. |  |
|  | **Ariel** | Telemedicine is helpful. |  |
|  | **Ella** | If there is no telemedicine, patients will have to go to [another province] just to consult with a physician. In telemedicine, they can already be prescribed medicines. |  |
| ***IN*** | *Don’t you find the English language hard to comprehend?* | |  |
|  | **Bella** | No, it is basic. |  |
| ***IN*** | *Did you attend other training sessions?* | |  |
|  | **ALL** | We attended the training area gender facilitated by [organization]. We also attend women’s summits. |  |
| ***IN*** | *Is that one time, Ma’am?* | |  |
|  | **All** | Yes. |  |
|  | **Fiya** | It is conducted yearly. |  |
| ***IN*** | *Aside from that?* | |  |
|  | **Ariel** | None so far. |  |
| ***IN*** | *Does anyone else want to share?* | |  |
|  | **Daria** | I hope UTD will continue so we can have free access and training since it really is beneficial for us. |  |
| ***IN*** | *Yes, Ma’am. For now, it will continue.* | |  |
|  | **Daria** | Yes, because of UTD, we are guided. |  |
| ***IN*** | *Would you recommend UTD use among primary care providers or fellow BHWs?* | |  |
|  | **Ariel** | Yes, so they can learn too. |  |
|  | **Cess** | So that we are not the only ones who can benefit from it. |  |
|  | **Bella** | So others like me can learn too. |  |
|  | **Daria** | I will share it with them so that when someone asks them, they know what to answer. |  |
|  | **Fiya** | Yes, so that it can be shared with family members who may need care. |  |
|  | **Ariel** | Yes, same for me. |  |
|  | **Ella** | I would recommend it especially in taking care of families and educating them about health cases so they themselves can do the necessary first aid treatment to themselves. UpToDate is called like it because it is always up to date. |  |
| ***IN*** | *Is there anything else you wish to share about your role, the challenges you encounter at work, and your views on UpToDate and the journal clubs?* | |  |
|  | **Daria** | Thank you to UpToDate as I was able to learn a lot. |  |
| ***IN*** | *So can you say that UTD has been effective in your work as a health care worker?* | |  |
|  | **ALL** | Yes |  |
|  | **Ella** | It really is a huge help. |  |
| ***IN*** | *Your feedback was very substantial and it shows how helpful UTD was in your profession. It’s also good to note the issues that hinder you from fully utilizing it. Alright, if you no longer have anything to share, this ends our FGD.*  *Again, thank you for attending!* | |  |

**--[End Transcript (01:14:26)]—**
