# Supplementary material for: Resilience, ingenuity, and identity: A multi-level analysis of the Filipino community health worker experience in rural and remote municipalities in the Philippines
Source: PLOS Glob Public Health. 2025 Aug 18;5(8):e0004965. doi: 10.1371/journal.pgph.0004965 (PMC12360505; doi:10.1371/journal.pgph.0004965)
Supplement: S1 File — (ZIP) [file pgph.0004965.s002.zip › 2023-07-09 PPCS GIDA Translation FGD 3.docx]

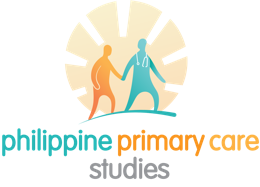


**Focus Group Discussion Transcription**

FGD 3 – Long Tenure

**Philippine Primary Care Studies**

NAST CHW Experience Study

**PRELIMINARY INFORMATION**

| Location: | Rural Health Unit of the Remote site in Southern Luzon |
| --- | --- |
| Date Recorded: | July 9, 2023 |
| Transcriber’s Remarks: | The informant’s name and key identifiers have been redacted from this transcription. |
| List of Acronyms: | NDP = Nurse Deployment Program  BHW = Barangay Health Worker  UTD = UpToDate  BNS = Barangay Nutrition Scholar  HRH = Human Resources for health  BHC = Barangay Health Center |

**TRANSCRIPTION**

**--[Begin Transcript (00:00:41)]—**

| ***IN:*** | *A while ago, you shared what you usually do during weekends, this time, can you share your tasks during weekdays as a BHW? Let’s start with Ma’am Elsa.* | |
| --- | --- | --- |
|  | **Elsa:** | [inaudible]…we sometimes conduct BP monitoring, checking of pregnant women and children, assisting in telemedicine, and other tasks in the barangay. |
| ***IN:*** | *Do you do house-to-house visits?* | |
|  | **Elsa** | Not daily, as needed only. |
| ***IN:*** | *On regular days, you check on patients. How about you, Ma’am Biel?* | |
|  | **Biel** | I usually do my reports first before patients come in. |
| ***IN:*** | *That’s your main task, Ma’am? Do you also have a duty? How many of you are in the barangay?* | |
|  | **Biel** | Just me. Since I am a BNS, my task is more on reporting. |
| ***IN:*** | *I see, Mam. But from time to time do you also attend to patients?* | |
|  | **Biel** | Yes, I help BHWs in attending to patients whenever available. |
| ***IN:*** | *Okay, Ma’am. How about you, Ma’am Cora?* | |
|  | **Cora** | In my case, Ma’am, I am the one who opens the barangay health center. Upon arrival, I clean the premises before the senior citizens for BP monitoring arrive. I also assist in conducting FBS for diabetic patients, however, we temporarily stop now due to a scarcity of supplies.  I also assist patients with referrals. We provide them with options for telemedicine or face-to-face consultation. They usually prefer F2F but when they learn that it is [doctor’s name] on duty, they resort to telemedicine since they are already familiar with the physician. They said that [doctor’s name] is hands-on as well and follows up after check-ups. |
| ***IN:*** | *I see. He really sees to it that patients are checked up even after the consultation.* | |
|  | **Cora** | We also monitor pregnant women for check-ups. We also receive reports from the neighborhood regarding new pregnant women in the community. This is important because some won’t report and we will be just surprised that she is 7 months pregnant and is about to give birth. |
| ***IN:*** | *How old is she?* | |
|  | **Cora** | She is a teenager, so she needs to be guided and monitored. If not for “marites”, we wouldn’t be able to know. Sometimes, people like them could be assets too. |
| ***IN:*** | *They really can be useful sometimes as they can be a source of information as long as it is factual of course.* | |
|  | **Cora** | Yes, you need to filter it too. |
| ***IN:*** | *So those are your usual tasks, right? Monitoring patients? Okay, who else wants to share? What are your daily tasks as BHW?* | |
|  | **Gina** | I start my day by cleaning the premises. Then if a patient comes for BP monitoring, I facilitate it. At 8 am, I contact pregnant women for check-ups and children for weighing. Sometimes, you have to convince the mothers to have their children monitored. Before they really won’t visit the BHC, we have to visit them at home, but now, they are more adherent and receptive. You just can’t avoid those who are lax about visiting. Even though I don’t have a schedule, I go to the center every day where I also assist telemedicine patients. We help each other in the barangay. |
| ***IN:*** | *It's good that you practice collaboration and cooperation in the center. How about you, Ma’am?* | |
|  | **Faith** | In our barangay, before I start with my duty, I attend to my [infectious disease program] patients first at the barangay hall since the barangay health center is a distance from the barangay hall. Some patients would opt to be consulted in the barangay hall due to its proximity to them. I also do BP monitoring of patients with hypertension and check lactating mothers. Once I arrive at the center, I do my reports, and then in the afternoon, we visit the patients who are unable to visit the center for weight monitoring, some of which live far from the población area. |
| ***IN*** | *You would really make an effort to visit.* | |
|  | **Danica** | In my case, Ma’am, I also do similar tasks such as BP monitoring, and visiting [infectious disease program] patients to distribute medicines, however, due to the scarcity of supplies, we can only provide limited medicines so we have to prioritize the indigent residents. I also do feeding activities, assistance to lactating mothers, and monitoring children’s weight. |
| ***IN*** | *I see, so these are your tasks for the whole day. How about you, Ma’am?* | |
|  | **Aya** | My duty is every Thursday but I see to it that I go to work every day. Previously, I was assigned to [another health area] monitoring but I got removed for some misunderstanding. As a BHW, you are expected to always be of service, whenever someone calls you, you should always respond no matter how tired you are. That is why balance and time management matter.  As much as possible, I do everything I cannot just for honorarium but because of my passion to serve. This is expected of you since you are in the public service. Sometimes, you will encounter issues, but life must go on. You just gotta do your work.  Thank you to primary care for the huge assistance not just for the free medicines, but most importantly the knowledge and learning you have imparted to us, especially doc dans whose experiences we can relate to. |
| ***IN:*** | *This is why we’re here today to continue to provide you with assistance.* | |
|  | **Aya** | Thank you to primary care. |
| ***IN:*** | *Thank you for sharing, Ma’am. I see a commonality in your answers in terms of your daily routine. Given that your group has the longest tenure in service, what do you think motivated you to stay?* | |
|  | **Aya** | It really is passion, Ma’am. |
|  | **Elsa** | It's good camaraderie with my fellow health workers. |
|  | **Biel** | I agree, what will you do with your work if you don’t like your companions? |
|  | **Faith** | It really is fun and fulfilling to serve the barangay. |
| ***IN:*** | *How about you, Ma’am Gina? How did you last 27 years?* | |
|  | **Gina** | I started as BHW in 1989 wherein being a BHW is just volunteer work. There was no honorarium and I had to visit the farthest households carrying a traditional weighing scale that is so heavy as it is made of steel. I love my job because I am able to help others not because of honorarium. |
| ***IN:*** | *So, didn’t you apply for other jobs?* | |
|  | **Gina** | What really attracted me to join the service was the training and seminars which are good avenues for me to learn new knowledge that I could apply to my family, not because of the incentive. There’s a time when my family wants me to resign but I still continue. |
|  | **Aya** | I really love taking care of patients especially the [infectious disease program] and diabetics for monitoring as they are very challenging to handle. We encountered all sorts of blood, phlegm, etc. |
| ***IN:*** | *It really takes a lot of getting used to it.* | |
|  | **Danica** | You need to have a strong personality. |
| ***IN:*** | *You are indeed a recipient of different kinds of people that’s why a lot of patience is needed.* | |
|  | **Biel** | Other patients think that we are already a hospital by demanding a lot of things from us. |
|  | **Danica** | When someone gives birth, all of the responsibilities are with you, even buying the diaper. |
| ***IN:*** | *And you use your own money for it? Being a BHW really isn’t an easy one. Perhaps your fulfillment in serving is one reason that motivates you.* | |
|  | **Gina** | Yes, Ma’am. Our salary was just 50 php before. |
|  | **Faith** | In my case, I had no honorarium for two months before. If there is, it is just as low as 150 php. |
| ***IN:*** | *It takes a lot of sacrifice indeed. Other than that, what else do you receive?* | |
|  | **Gina** | It was just now that I experienced a higher salary that’s why I always tell my workmates not to focus on the honorarium but rather on the work they’re doing. You need to love it. |
| ***IN:*** | *How about you, Ma’am Elsa?* | |
|  | **Elsa** | I learned to love my job. Previously, I resigned from being a BHW to look for a better opportunity in Manila as a tailor; however, tho I was earning okay, I am still yearning for my work as a BHW. And so when I came back, I never left again because I already love what I am doing. |
| ***IN:*** | *It seems like there is no other job that can give you a sense of fulfillment than of being a BHW. How about you Ma’am Biel?* | |
|  | **Biel** | We’re okay in [our barangay]. There are seldom additional tasks. I lasted for so long due to my workmates. It really is more productive to be doing something rather than staying at home. |
|  |  |  |
| ***IN:*** | *You should consider training potential BHWs, Ma’am so someone can replace you when you retire.* | |
|  | **Biel** | Only a few want to pursue the job these days since they assume that there is an overload of work for a very minimal pay but we actually only get busy during January and July. And every December, we also receive incentives. |
| ***IN:*** | *Incentives are great motivators. Is there anyone who wants to share your reasons for staying as a BHW? As I can see, most of you have similar answers such as a passion for service, a relationship with workmates, and comfortability in the working environment. These things play a huge part in your job. How was your working environment at the barangay level?* | |
|  | **Aya** | I’ll start with the barangay officials; they are very supportive and accommodating. In terms of relationships with other people, I must say you really cannot please everybody, that’s why you need to be firm in what you need to do. As a BHW, I am strict because I know what I am doing. I will do my job even though some will disagree. In any barangay, there are those who are cooperative and there are those who are problematic. This is the very reason why I can’t leave my job because I’m worried about who will take care of the patients if some of the staff are too passive. Things get hard sometimes but I just pray for strength to serve the people because when you serve the people, you are also serving God. |
| ***IN:*** | *That’s correct, Ma’am. That plays a huge impact on you to stay long in the job compared to others with a toxic environment, they usually feel that they are no longer growing. How about you, Ma’am?* | |
|  | **Danica** | In our case, it is all good too. The barangay council supports our activities. My colleagues understand each other and promote cooperation. We share our duties and cover for each other especially when someone falls short. |
| ***IN:*** | *Okay, so that is your strategy. I do hope you train or mentor your staff as well. How about you, Ma’am?* | |
|  | **Faith** | I have no problem with my colleagues, only with the barangay officials. But I always tell myself to just do my job nevertheless. We feel like we are not being supported enough especially when there are visitors from RHU. It’s really hard to work if you are not well-supported. |
| ***IN:*** | *That’s true, Ma’am. It also limits our ability to serve others.* | |
|  | **Faith** | We were able to survive tho. We just don’t stop, especially in promoting health education. We will do everything we can for the betterment of our community. |
| ***IN:*** | *Especially since residents usually go to BHCs first as their first point of access,* | |
|  | **Faith** | Our common problem is that the medicines are being managed by the barangay council and kept in the barangay hall instead of the barangay health center due to the barangay captain assumes that the medicines are missing in the center when in fact, nothing is missing but rather they were being distributed to the patients. Ma’am [supervisor] also conducts inventory weekly. This is a problem because when a patient visits the center and needs medicine, he/she has to go to the barangay hall, and it has a distance from the center. |
| ***IN:*** | *So instead of medicines being stored in the center, they are rather kept in the barangay hall?* | |
|  | **Faith** | Yes, Ma’am. There are times that it gets hard but as I always say to my colleagues, we just continue to fight and do our work for the sake of the community because the people need us. Our problem though is the scarcity of supplies and resources needed especially for emergency cases. I have already requested the barangay but we are not being prioritized. If only we had the full support from the barangay, we could provide better service to the beneficiaries. A barangay is prosperous if it focuses on the health of the people. I sometimes feel like resigning but people are relying on me. That's why I have to keep going. These are some of the things that motivate me. |
| ***IN:*** | *It really is ideal if the leader prioritizes health programs. If the people are healthy, the country has a productive workforce as well. How about you Ma’am Gina, how is your working environment?* | |
|  | **Gina,** | For now, I am good with my work. The only thing I wish though is for the barangay council to take action and support the health programs such as the provision of vitamins especially to undernourished children. Every session, I always push for it, however, no action is being taken. I want them to experience our work on the ground. If there’s a problem, they should be able to help. I did not fall short in reminding them. |
| ***IN:*** | *Communication is important; however, they should be receptive too.* | |
|  | **Gina** | Another problem is the Kagawad on Health which is ineffective and not functional. |
| ***IN:*** | *What made you stay tho?* | |
|  | **Gina** | I just do my work even without their help and support. |
| ***IN:*** | *I see. You just do your job, right? Anyway, it’s the patients as your clients that you should please. How about you, Ma’am Cora?* | |
|  | **Cora** | Our working relationship among the BHWs in our barangay is good. My only concern is whenever RHU asks for a report with a very tight deadline, not considering that each BHW has a different level of understanding, especially those who are older and not technology savvy. That’s why I sometimes help them too and fill in their lapses. They try their best to adapt and comply as well. We don’t have resources either; I even use my own flash drive. I hope when asking for reports, ample time can be given.  Since the pandemic, everything has been mostly via messenger. That’s why most of the BHWs really saved to buy a phone and some even have to borrow money. As someone who knows how to use these devices, I teach them how to use them as well as how to print referrals and other documents. |
| ***IN:*** | *So you have cited the problems you have encountered, other than those mentioned, is there anything you would like to add? How about you, Ma’am Biel?* | |
|  | **Biel** | I have no problem with my barangay council since they are supportive of my requests. I told them that they should not give us a problem since the barangay reports depend on us.  My only concern is handling hard-headed patients such as those with hypertension who don’t want to visit the center. Instead, they want to be visited in their houses even though they are not PWDs and capable of visiting the BHC. It just so happens too that our BHW is also a PWD.  Also, I hope we don’t get rushed in submitting reports too. |
| ***IN:*** | *I understand that, Ma’am. As HCWs, your focus should be on the patients only, however, there are administrative tasks too and it really is hard to manage at times.* | |
|  | **Danica** | Good thing if the reports are easy to prepare and can be done in the rush but it is the other way around. |
|  |  |  |
| ***IN:*** | *How about you, Ma’am Elsa? Would you like to add?* | |
|  | **Elsa** | In our case, there is not much of a problem in terms of health activities since we are well-supported in terms of supplies in the barangay. In the health center, we have a good relationship with our midwife. My only issue is the other BHWs whom I feel lack respect and don’t perform well having no initiative even with cleaning the premises. What I don’t like about work is when people take things personally especially since I’m just doing it for the barangay and not for my name. We should be helping each other and data requested for the reports must be complied with on time by the BHWs. |
| ***IN:*** | *How do you handle those, Ma’am?* | |
|  | **Elsa** | I just do my job, up to you, if you won’t follow. Others are okay and are very active in the activities. |
| ***IN:*** | *Alright. Thank you for sharing. Now, What resources do you believe would better support you in fulfilling your responsibilities? You mentioned the BP apparatus a while ago, what else do you think you need?* | |
|  | **Cora** | Laptop, Ma’am but instead we were provided with a desktop. The problem with the desktop is you can’t bring it to seminars and can’t use it during brownouts. |
|  | **Biel** | Cellphone. |
| ***IN:*** | *Especially in preparing the data and reports, right* | |
|  | **Danica** | In our barangay, we have two desktops, however it got broken due to the intermittent brownout. |
| ***IN:*** | *Devices are really of value in your line of work as BNS.* | |
|  | **Aya** | Yes, Ma’am, the same with the printer. Our barangay secretary has a printer which we use from time to time as well but it is not sufficient since the barangay council is using it too and our reports usually take a lot of pages. |
| ***IN:*** | *I see that. It seems like everyone shares the same kind of resources they need as all of you are BNS. So a while ago, you mentioned the different challenges and hindrances you have encountered in your job such as lack of supplies, relationship with colleagues, and non-adherence of patients.* | |
|  | **Danica** | My husband is already complaining since I am always at work and I go home late at night.. |
|  | **Faith** | Don’t replace your husband with your work. |
| ***IN:*** | *That’s true, Ma’am. You should keep things in balance. Your husband is probably just concerned about your welfare and with you being overworked.* | |
|  | **Faith** | My husband on the other hand is supportive of my work and he even does errands for me such as distributing medicines since he has a tricycle. |
| ***IN:*** | *That's good that you are being supported by your husband.* | |
|  | **Faith** | Yes, good thing too that he has a tricycle |
| ***IN:*** | *Yes, and the fact that he understands your job especially since we know how demanding our work can be.* | |
| ***IN:*** | *So far, do you have any challenges encountered that you are yet to share? Or that’s all?* | |
|  | **Cora** | May I add, Ma’am? Another issue is the entitled patients, especially those for HPN monitoring. Sometimes they are hard to convince as they feel like they are more important than the other tasks that you were given to accomplish. |
| ***IN:*** | *A lot of understanding and patience is really needed. You need to go over it again and again. .* | |
|  | **Cora** | Others will even get mad at you especially if you can provide them with medicines without even thinking that we only have limited ones. That's why we often tell them to just go to the pharmacy and buy their own. |
|  | **ALL** | That’s true. |
|  | **Danica** | Others get mad too if there is no physician and it’s even beyond our control. |
|  | **Cora** | Sometimes, the barangay council decides on the health center even though some are health-related concerns such as managing medicines. They usually distribute it to patients even though it wasn’t prescribed or they don’t really need it without consulting a physician or HCW. |
|  | **Faith** | Such as antibiotics, some patients decide on their own as to the amount and duration of taking as if they know more than you. If you reprimand them, they will get mad at you. |
| ***IN:*** | *That is correct, Ma’am. That’s why you really need to be firm and strict since it is for their benefit anyways.* | |
|  | **Gina** | There is one patient that told me I am so selfish for not providing her with the medicine she doesn’t need or not prescribed. I confronted her and made her understand. I told her that I am just doing my job and it is my duty to ask for a prescription when dispensing antibiotics. I don’t want to dispense medicine that I know may cause harm to anyone especially if not prescribed. |
| ***IN:*** | *That’s correct, Ma’am. We need to be strict since we are the ones who truly know as HCWs. If they do get mad at least we just did our job and what we’re doing is for the welfare* | |
|  | **Gina** | People do have different attitudes and personalities. |
| ***IN:*** | *That is really a part of the challenge we face every day. Okay then, let’s proceed to the next question. How do you feel about the use of technology or medical software for your scope of practice? Does it help you in your work and makes you an effective BHW?* | |
|  | **All** | Yes, it is a huge aid. |
|  | **Danica** | Yes, Ma’am. A huge help. |
| ***IN:*** | *In what way, Ma’am?* | |
|  | **Aya** | When it comes to knowledge and information. At first, I thought Primary Care is just a bill we are trying to pass but now I realize that it is for practical application that can improve our health system. It is not only suited for the family but it covers a lot of health areas and topics that are very helpful. This is why we are thankful to [organization] and UHC. I really do hope it gets pushed through. |
| ***IN*** | *We are waiting for that to happen as well. [Doctor] and [organization] really continue to push for it and be implemented soon.* | |
|  | **Aya** | But it isn’t a priority of our President though. |
| ***IN*** | *DOH has a limited budget too.* | |
|  | **Danica** | UTD actually is the most integral part of our job as HCWs. |
| ***IN*** | *Who else shares the same opinion?* | |
|  | **Gina** | UTD is a huge help, especially in gaining knowledge about health that we can use in our work. |
| ***IN*** | *Are you comfortable using it? Is it user-friendly? It is actually similar to searching in Google so even those who are not tech-savvy can adapt.* | |
|  | **Gina** | Yes, Ma’am. I am able to follow it since it is easy to use. If there are things I don’t know, I just ask my colleagues. |
| ***IN*** | *Do you usually use your phones to access them?* | |
|  | **ALL** | Yes, Ma’am since it is easier to use. |
| ***IN*** | *How often do you use UTD?* | |
|  | **Cora** | Sometimes, when I am bored, I find myself casually reading it. I just search for random topics and read. |
|  | **Faith** | When I need something to learn about a case, I just look it up at UTD. |
|  | **Cora** | When there are patients who would like to have their laboratory results interpreted, I often check UTD to define the terminologies but I also refer them right away to the physician for proper assessment. There are times too that I find it hard to understand some of the terms and topics in UTD so I make sure to be careful in advising patients. |
| ***IN*** | *Yes, Ma’am. That is important so that they get properly diagnosed. Other stuff is too technical even if you search it in UTD.* | |
|  | **Aya** | I agree. That’s why I attend the journal club because I gain a lot of insights from it such as handling PMS which is very common. |
|  | **Biel** | That’s also why UTD is so nice because whenever someone will consult, you can easily refer to it, and just refer them to the physician if a higher level of care is needed. |
| ***IN*** | *So far, based on what you have mentioned, the feedback for UTD is positive as all of you see it as helpful too in your daily work. How do you find the journal club every Thursday?* | |
|  | **Danica** | It is nice although we don’t always attend due to bad signal or if we have prior work commitments. |
|  | **All** | Our signal is weak. |
| ***IN*** | *The signal is indeed the no. 1 problem. But how was your experience so far in the discussion? Was it helpful and easy to understand?* | |
|  | **All** | It’s good and we were able to share our thoughts and ask relevant questions. We also get to meet other BHWs in Samal whom we also learn from. |
| ***IN*** | *Who among here has already experienced presenting in the journal club? How was it?* | |
|  | **Danica** | My colleagues were able to share during one of the discussions. It went well and they felt happy and fulfilled sharing as it boosted their confidence especially since they do not expect how many the audience was. The only issue is the erratic signal which hampers the presentation. |
| ***IN*** | *Again, that is the challenge. Now, moving on to the next. What do you think are the benefits and disadvantages of using UTD in your work?* | |
|  | **Danica** | In my case, Ma’am. UTD helps in boosting my morale and confidence. As a BNS, people would undermine our work, but little did they know that we know more than they do in terms of health management. |
| ***IN*** | *It empowers you.* | |
|  | **Aya** | Yes, Ma’am. I don’t say that because I want to come off as boastful or what but it is what I feel whenever some people would degrade us. They have no idea that we have regular training with certificates, thus we have sufficient knowledge too. |
| ***IN*** | *The good thing about it is that when patients ask you, you can confidently answer and explain it to them, thus earning their trust and establishing your credibility.* | |
|  | **Danica** | Yes, even though we are just BHWs, we know things and we won’t let other people undermine us. |
|  | **All** | Yes. |
|  | **Gina** | We may be more knowledgeable than you. |
| ***IN*** | *You are lucky to have free access among all municipalities in Sorsogon and the Philippines.* | |
|  | **Biel** | Good thing really that we have access. |
| ***IN*** | *Except for journal clubs, do you have other training attended?* | |
|  | **Aya** | Actually, Ma’am, our training was put on hold during the pandemic but we are more than willing to be trained because it is what we really need. |
| ***IN*** | *Okay,* | |
|  | **Cora** | We have one training in [a medical topic] in RHU. It’s training relative to the profiling system. However, it has so many errors and thus cannot be implemented yet. |
|  | **Danica** | So far, we don’t have any training right now. |
| ***IN*** | *So aside from EMR or UTD, you also have other system applications introduced to you. We are really heading towards digitalization.* | |
|  | **Cora** | Same with a digital vaccination certificate. It is paperless. |
| ***IN*** | *We are in a digital age indeed.* | |
|  | **Cora** | The only concern is I don’t have a laptop and there are many reports required of us in a limited time yet we lack the resources. |
| ***IN*** | *That’s a reality, Ma’am.* | |
|  | **Biel** | Other barangays don’t even have a supply such as bond papers. |
|  | **Danica** | That is true. That’s why it is so hard to comply. |
| ***IN*** | *It is a fact, Ma’am. That happens.* | |
|  | **Cora** | We are the ones in the ground and the ones sacrificing and yet they expect us to have everything figured out at a very tight deadline not considering that each BHW has a different level of understanding. |
| ***IN*** | *What do you think can still be improved in the journal club?* | |
|  | **Cora** | It would be better if we are guided in preparing and discussing our presentation and give us ample time to prepare. We also get a little too nervous presenting. |
|  | **Aya** | We still lack exposure and confidence. |
|  | **Danica** | We need a little more practice. It would be better if someone would guide and mentor us. What happens is the one who prepared the slides is different from the one who will report; thus the discussant finds it hard to present it since he/she is not familiar with it. This is why presentations should be given to the presenter so he/she can have enough time to familiarize herself. |
| ***IN*** | *Ideally, you really should be guided by the HRH.* | |
|  | **Aya** | *It should be reviewed step by step. Sir [administrative officer] used to mentor us on how to do it so we are guided once we present.* |
|  | **Cora** | It’s embarrassing that some participants from [our province] could not answer when asked by [doctor] since he/she doesn’t know well the topic being presented |
|  | **Biel** | Other BHWs should help when that happens.  We also have one concern regarding the journal club attendance. Some of us have a hard time accessing the Google form and inserting proof of attendance such as uploading photos or screenshots. The problem is we only use one laptop for all of us however the Google form requires an email for each, and thus not all have the device to access it. |
| ***IN*** | *Alright, Ma’am. Noted. We will let the facilitators know.* | |
|  | **Gina** | It’s also a hassle if you are not that techy. |
| ***IN*** | *Alright, Ma’am. Noted. Is that all you can think of for the points of improvement for the journal club? With this, would you recommend the use of UTD?* | |
|  | **ALL** | Yes. |
| ***IN*** | *Why is that so? Let’s start with Ma’am Elsa.* | |
|  | **Elsa** | I can share my knowledge with others. |
|  | **Biel** | Yes, since it is helpful especially when patients consult. Also, I mentor the other BHWs on how to use it so they can maximize it as a reference. |
|  | **Cora** | I will recommend it since you can download it offline especially when you don’t have a stable internet. If you have questions, you can just search it and it will give you the things to do, avoid managing such cases, and when to seek a physician. |
| ***IN*** | *Yes, Ma’am Gina?* | |
|  | **Gina** | Yes since I find it useful whenever someone will ask me, I use it as a reference. |
| ***IN*** | *I am actually amazed at how you can adapt despite your age. This is proof that if you really want it, nothing can hinder you from learning.* | |
|  | **Biel** | The only issue is the signal. |
| ***IN*** | *The Internet plays a very important role indeed.* | |
|  | **Faith** | It is helpful especially that we continue to add BHWs in our barangay. We teach them how to navigate and use it as a reference. |
|  | **Aya** | It is beneficial not just to you or your family, but also to others. |
|  | **Danica** | What’s good about UTD, is that it is detailed and comprehensive that even a regular patient can understand it. |
| ***IN*** | *Yes, Ma’am. The application offers options for different levels of understanding, from patients to physicians. You can also translate it into Tagalog. I can see that UTD has been really beneficial to you, especially in furthering your skills and knowledge about certain cases.*  *Is there anything else you wish to share about your role, the challenges you encounter at work, and your views on UpToDate and the journal clubs?* | |
|  | **Aya** | Lastly, I hope that [organization] or [doctor] will continue to push through the implementation of UHC because that is what we really need. Things are getting hard these days and what we need is a good health system. |
| ***IN*** | *I really hope so too, Ma’am. Actually, as part of pushing for a good health system for all, this study is one initiative of primary care that aims to improve the quality of care and push for policies that are aligned with the needs of the workers on the ground.*  *You have a big role to play and so we are very thankful for your time and cooperation in joining us today.* | |

**--[End Transcript (01:34:19)]—**
