# Supplementary material for: Resilience, ingenuity, and identity: A multi-level analysis of the Filipino community health worker experience in rural and remote municipalities in the Philippines
Source: PLOS Glob Public Health. 2025 Aug 18;5(8):e0004965. doi: 10.1371/journal.pgph.0004965 (PMC12360505; doi:10.1371/journal.pgph.0004965)
Supplement: S1 File — (ZIP) [file pgph.0004965.s002.zip › 2023-07-09 PPCS GIDA Translation FGD 1.docx]

**Focus Group Discussion Translation**

FGD 1 – Short Tenure

**Philippine Primary Care Studies**

NAST CHW Experience Study

**PRELIMINARY INFORMATION**

| Location: | Rural Health Unit of the remote site in Southern Luzon |
| --- | --- |
| Date Recorded: | July 9, 2023 |
| Transcriber’s Remarks: | The informant’s name and key identifiers have been redacted from this transcription. |
| List of Acronyms: | NDP = Nurse Deployment Program  BHW = Barangay Health Worker  UTD = UpToDate  BNS = Barangay Nutrition Scholar  HRH = Human Resources for health  BHC = Barangay Health Center |

**TRANSCRIPTION**

**--[Begin Transcript (00:00:15)]—**

| ***IN:*** | *The first thing I will be asking has something to do with your role as BHW. These are the things or tasks that are assigned to you in the barangay. What are these responsibilities?* | |
| --- | --- | --- |
|  | **Bea:** | As a BHW and a [an affiliate in an international infections diseases program], what I do in the morning is usually check patients and monitor the PWDs if there are any concerns that they want to relay to Ma’am Sally (HRH). |
| ***IN:*** | *Is Ma’am Sally your supervisor?* | |
|  | **Bea:** | Yes, she is our supervisor for PWD management. And then if there is a concern, I am the one who relays it to the physician. I also assist in the provision of ID to the PWDs in our barangay. During the month of December, I oversee checking and consolidating the list of PWDs who will be receiving assistance such as a grocery pack. We make sure that only those who are considered indigent are prioritized as the recipients. This is also one way of convincing others to register.  I also source medicines from the center to be distributed to patients with hypertension, however, we only have a limited supply of medicines. I also do rove and visit patients just to distribute the medicine and ensure that they can take it. Some of the patients will ask or demand more but we could not provide since we are short of supply. I make them understand instead that since there is a scarcity of medicine, we should learn how to share it with others who need it as well. What matters is that they have at least some medicine to take.  Being a concerned citizen, you don’t just serve your family but also your fellow community. You will do whatever you can to help. |
| ***IN:*** | *It’s like you already have a routine every day.* | |
|  | **Bea:** | My schedule is already planned from Monday to Friday. |
| ***IN:*** | *So it’s like there are specific activities for each week?* | |
|  | **Bea:** | Yes, Ma’am. Since I have to work every day. And aside from that, I also take care of the elderly. |
| ***IN:*** | *Aside from taking care of PWD, you are also tasked to look for elderly people.* | |
|  | **Bea:** | No, Ma’am. Just my elderly husband who also is a PWD. My children are still young thus they are unable to take care of my husband. I am the only one who manages him. I have full-time work in the morning, then at night, I take care of my husband. That is why I always ask permission from the barangay captain to allow me to go home early. |
| ***IN:*** | *All right, Ma’am. Thank you. Who else wants to share? What are your responsibilities in the barangay as health workers?* | |
|  | **Emma** | I usually assist patients who visit the center for BP Monitoring. |
| ***IN:*** | *Oh okay, so you also check patients as well?* | |
|  | **Emma** | Yes, whenever patients visit. |
| ***IN:*** | *Who is your supervisor currently? Other than that, what else are your tasks?* | |
|  | **Emma** | Ma’am Yel is my supervisor. I also do administrative tasks such as printing reports or referral forms. |
| ***IN:*** | *Aside from that, Ma’am?* | |
|  | **Emma** | When I monitor patients. |
| ***IN:*** | *Do you also go house to house for monitoring?* | |
|  | **Emma** | No, not really. Only when patients visit the BHC. |
| ***IN:*** | *Okay, Ma’am. How about you, Ma’am Cath?* | |
|  | **Cath** | We have a schedule for our weekly duty. Each one of us has a task. Mine is the distribution of medicines to those with maintenance. The Kagawad on Health is the one managing the supplies and inventory. I just assist her. I also conduct house-to-house monitoring of patients with hypertension.  Just the other day, I handled a patient who was bitten by a snake. The patient went to BHC to seek help and we immediately conducted a first aid treatment before referring the patient to RHU. |
| ***IN*** | *I see. The referral system is still being practiced* | |
|  | **Cath** | Yes, patients are already oriented that they should consult in BHC first before going to RHU for a referral. |
|  |  |  |
|  | **Faye** | In our case, we are five BHWs in all. Tuesday is my duty. I go to work as early as 7:30 am after tending to the needs of my family. My task is monitoring the patients with hypertension and getting their BP. I handle [a certain area in our barangay]. I also handle emergency cases that’s why as BHW, we are expected to be always alert even at night. |
| ***IN*** | *The work of a BHW doesn’t really end at 8:00 am – 5:00 pm, right?* | |
|  | **Faye** | All of us BHWs also augment during blood donation, vaccinations, and other health activities in the community while every Sunday we participate in communal gardening along with the Barangay councils. As BHW, you are bound to do a lot of tasks other than the scope of your work. |
| ***IN:*** | *That’s really part of your work as a healthcare worker and at the same time, a frontliner. You are indeed expected to always be on alert. You have also mentioned that you have a duty every Tuesday, what do you usually do during that day?* | |
|  | **Faye** | We also sometimes do administrative tasks such as reporting especially during accreditation. |
| ***IN:*** | *Your roles are really important as healthcare workers. How about you, Ma’am Dane?* | |
|  | **Dane** | In our case, Ma’am. There are five BHWs in the barangay. Each of us has his/her own duty as well. Whenever I’m on duty, I do BP Monitoring of patients. Sometimes, we are also asked to augment in preparing reports to be submitted to RHU. Also, we attend the Thursday UTD club. Our only problem is the low signal in our barangay. We try our best tho to still attend despite the hindrance. We usually connect to the barangay hall because they have a wifi connection. |
| ***IN*** | So, do you really go to the barangay hall to connect? Signal and wifi connection are indeed a common challenge to everyone. But I’m glad that you are still able to attend nevertheless. | |
|  | **Dane** | Yes, Ma’am. We really do try our best to join the journal club. |
| ***IN:*** | *All right, Ma’am. How about you, Ma’am Anna?* | |
|  | **Anna** | As a BNS, my task is more on reporting and consolidating data. My duty is every Thursday wherein my usual task is conducting BP monitoring of patients with HP, getting vital signs of patients, and referring patients to RHU. I also assist in facilitating telemedicine. I am also assigned to live birth. |
| ***IN:*** | *That is your main task in the barangay?* | |
|  | **Anna** | Yes. |
| ***IN:*** | *I’ve also observed that almost everyone has common daily tasks as BHW.* | |
|  | **Cath** | I also help in conducting [a national nutrition program implemented in our barangay], Ma’am. That includes monitoring the weight of children. This is done for a whole day in the barangay. |
|  | **Faye** | In our case, we do the weighing of children in the center only since we don’t have a weighing scale that is portable or easy to bring for a house-to-house visit. |
| ***IN:*** | *I see. How about the others?* | |
|  | **Cath** | We do house-to-house visits, Ma’am starting from upper to lower barangays. |
| ***IN:*** | *Okay so based on what you’ve said, your task includes checking patients, monitoring their BP, and doing counseling sessions. What do you usually counsel?* | |
|  | **Cath** | Family planning, Ma’am. We also manage issues relating to marriage especially those who have issues with pregnancy. We also handle VAWC. We assist the kagawad on health. |
| ***IN:*** | *It really does happen, Ma’am. Did you undergo training for handling such cases?* | |
|  | **Cath** | Yes, we attended a seminar. |
| ***IN:*** | *That’s good since such topics are sensitive and need proper guidance. Is there anyone else who wants to share? I guess none.*  *Now, you mentioned a while ago your responsibilities in the barangay. The way you shared it, it seems like you feel happy and satisfied in your work, if I assume it correctly.*  *.* | |
|  | **Faye** | Sometimes we do feel stressed too to the point of thinking of resigning. |
|  | **Anna** | Yes, that’s true sometimes. Especially if we are burned out of deadlines. |
| ***IN:*** | *I see. So that’s one of the many challenges, right? Meeting deadlines and at the same time handling patients too. It is a common health problem where health workers have overlapping tasks wherein they should be focusing on the patients only supposedly thus affecting the quality and efficiency of service.* | |
|  | **Cath** | We also sometimes assist the visitors who conduct house-to-house surveys since BHWs are usually the ones familiar with the houses. |
| ***IN:*** | *What made you then stay as a BHW despite these challenges?* | |
|  | **Faye** | For me, it’s really the learning gained from it and being able to socialize with other people. Although it could be stressful at times, there is still a sense of joy. |
| ***IN:*** | *So what really motivated you to stay at BHW is the knowledge you are gaining that could you’re you develop both professionally and personally.* | |
|  | **Faye** | Yes, and we could share it with our families as well. |
| ***IN:*** | *These are the so-called benefits of BHWs. Other than that, what else can you think of?* | |
|  | **Faye** | Before, I really didn't care about getting involved in the barangay as I was focused on my family, however, eventually I feel like I am doing nothing productive and so I started getting active in the community and became the president of [organization] and served as [special committee] Officer. Socializing helped me a lot to gain experience and expand my network. |
| ***IN:*** | *It really did help you grow and develop yourself. Is there anyone else who would like to share?* | |
|  | **Dane** | As a BHW, it really is a challenge to deepen my learning, which is why I also enrolled in [a government vocational program]. I also make sure to attend UTD since I am able to use it in doing my job and at the same time, I feel engaged in learning new things.  Entering the work as a BHW is indeed challenging. I usually spend my spare time googling health-related cases and learning about them so I can serve the patients and the community better so that more patients will seek help from us especially since I am still a little bit new as a BHW. |
| ***IN:*** | *It is good that you really initiate learning and be able to use that learning to help others. How about the others?* | |
|  | **Cath** | Other than that, I am also able to help my family and other people, especially the children, other feeding programs, and pregnant women. There are times that I want to quit already due to the problems and issues in the barangay especially if your work is not being recognized. Those are some of the disadvantages as well but overall, I am okay with my job especially since I have a good relationship with my fellow BHWs, although sometimes, the “marites” culture is rampant. Other than that, my supervisor is nice and accommodating. |
| ***IN:*** | *The relationship you have with your colleagues indeed plays a huge part. If you feel comfortable in the workplace, then it will help you be more motivated in your work* | |
|  | **Cath** | Sometimes, problems and conflicts arise too especially in handling patients or constituents during assistance aid distribution where everyone feels entitled. You will be the one to blame when some names are not included in the list. |
| ***IN:*** | *How do you handle those problems, Ma’am?* | |
|  | **Cath** | I try to explain and make them understand so they would stop complaining. Such things are already part of the challenges. |
| ***IN:*** | *That’s why as health workers, we should really know how to handle people. That requires a lot of understanding and patience. We encounter different people every day with different personalities too. How about you, Ma’am Jean? What motivates you?* | |
|  | **Emma** | [inaudible] I am able to learn more, especially in doing BP monitoring and proper management of patients. |
| ***IN:*** | *Aren’t you a new BHW? Do you think you feel like you would last more years?* | |
|  | **Emma** | Yes, especially since I am able to help people which I was not able to do before when I wasn’t yet a health worker. |
| ***IN:*** | *What made you join the health care work then?* | |
|  | **Emma** | I like taking care of patients, especially children |
| ***IN:*** | *I see. How about you, Ma’am Bea?* | |
|  | **Bea** | It was not easy at first, I am very shy when interacting. I was usually scolded as well, even though it was not my fault. I used to cry a lot but as time went by, I learned to be more patient and understanding.  When I entered BHW work, my husband suffered from a stroke. The knowledge I acquired from being a health worker helped me properly take care of and manage my husband such as monitoring his BP, administering his medication, etc. Also, whenever I am working at the center, it is a relief and stress reliever for me since some of the patients are nice to interact with. This somehow boosts my morale,  It would not be avoided that there will be workmates that you will not get along with but I don’t mind it that much. My confidence was also boosted little by little. Things like that don’t affect me that much anymore. What matters is I just do my job and help others in need, not just my family but also my friends through service, especially if there is assistance or aid from the barangay, I usually refer to them as recipients. I also assist in the community pantries.  I was a teacher previously in Manila but when the school closed, I really felt sad. That is when I went back to [my province] and continued serving. As a BHW, it really is a huge help as I was able to prolong my patience and deepen my understanding. I am able to blend with different types of people too. |
| ***IN:*** | *That really is one of the many learnings and that you are not just being able to help people but also yourself. Thank you, Ma’am. How about you, Ma’am Anna?* | |
|  | **Anna** | For me, it helps boost my confidence especially when we do house-to-house visits and we are able to meet and interact with people who seek help from you, giving you a sense of value and trust.  There are also activities where we can gain knowledge that is useful in my work. |
| ***IN:*** | *Okay, anyone else wants to share? Being a BHW makes us proud. Though it is not usually highlighted, BHWs play a huge role in managing patients since you are the ones who are on the ground and who serve as frontlines. That is what [organization] is trying to push forward, to provide you with training opportunities that will further enrich your capacities through UTD.*  *I mentioned a while ago that one of the things that motivates you to stay in your environment and have a good relationship with your workmates. Do you think a good working environment with a good supervisor matters for you to be more productive?* | |
|  | **All** | Yes |
| ***IN:*** | *Why do you think so?* | |
|  | **Bea** | A good relationship with your leader and workmates matters because the productivity and efficiency of work are affected. You won’t be able to deliver those reports, if you are not on good terms or if teamwork is not okay. A good relationship promotes mentoring as well especially since not all were able to graduate. If you are someone who is knowledgeable then it is your duty to help them.  Plus if there are emergencies, you can help each other to accommodate it. If the working relationship is not okay, then nothing will be delivered, that’s why maintaining that relationship with workmates and supervisor does matter. |
|  | **Anna** | Also, as a BNS, I find it hard to consolidate reports since some of the BHWs are not submitting on time due to different priorities. |
|  |  |  |
|  | **Cath** | Yes, it really depends. Good thing our supervisor is considerate. Even if we sometimes submit late or find it hard to comply, he/she will assist us. It really is important to maintain that kind of relationship. |
|  | **Faye** | What is important for me is respect for each other. Whenever we fail to comply on time, our BNS helps us instead especially if we are loaded with work. We should not just help each other but also respect one another. |
| ***IN:*** | *Alright. Who else?* | |
|  | **Dane** | I am happy with my supervisor (BNS) as a new BHW, she guides me especially when I am handling a big number of households. She is the one guiding me on what to do. |
| ***IN:*** | *It's good that you are being mentored by your supervisor.* | |
|  | **All** | Yes, Ma’am. |
| ***IN:*** | *Okay. Anyone else want to share?* | |
|  | **Emma** | Same with me, Ma’am. It is also a good relationship. |
| ***IN:*** | *Since you are still a new BHW, how do you find your workmates?* | |
|  | **Emma** | Also the same with others, Ma’am. |
| ***IN:*** | *Do you have any problems with your supervisor? Are you being mentored? Are you comfortable?* | |
|  | **Faye** | We treat each other as family. |
| ***IN:*** | *Everyone is equal indeed.* | |
|  | **Anna** | Yes, Mam. The only challenge for me as a supervisor or one that handles BHWs are the older workers, I sometimes feel shy or aloof in following reports from them. |
| ***IN:*** | *I see. So it is a challenge for you the fact that you are handling BHWs who are more tenured and older in years. How do you manage this?* | |
|  | **Anna** | I make sure to treat them with respect whenever I am asked for reports. |
| ***IN:*** | *That is a good approach, Ma’am. What do you think are the other resources that you need to help you with your work?* | |
|  | **Cath** | Improvement of the signal since we don’t have an internet connection especially when you are on duty and you need to be contacted. Also, you need an internet connection for UTD and for referring patients in telemedicine. You also need it, especially for emergency cases. |
|  | **Faye** | Same with us, Mam. |
|  | **All** | Also, with us. No wifi. |
|  | **Anna** | No load and wifi. |
|  | **Cath** | In our case, the barangay health center is separated from the barangay. The barangay hall is the one with wifi. We still need to go to the barangay to connect. |
| ***IN:*** | *So how do you contact me during emergencies?* | |
|  | **Cath** | We usually ask tricycle or habal-habal drivers to relay the information or we have no choice but to really go there. |
| ***IN:*** | Aside from the internet, load, what else do you need? | |
|  | **Anna** | Perhaps, additional medicine supplies such as thermometers. Other equipment purchased is easily destroyed. |
|  | **All** | We really do lack medicine supplies. |
| ***IN:*** | *Is it the barangay who purchased that?* | |
|  | **Anna** | Yes, depending on the budget. It would be better if we have our own apparatus so we can use and bring it to house-to-house visits or during emergencies. |
|  | **All** | That’s correct. |
| ***IN:*** | *That’s a good suggestion of having each of you have your own apparatus.* | |
|  | **Anna** | Yes, but it really depends on the budget of the barangay. |
|  | **Faye** | I really want to have our own wifi in the center so we can maximize telemedicine and UpToDate. |
|  | **Cath** | The problem is that our barangay hall is far from our center. The wifi is in the barangay hall only. |
| ***IN:*** | *Yeah, it would have been ideal if the two are adjacent to each other, or if not both should have a connection of their own. So these are now the challenges. Other than that, is there anything you want to add?* | |
|  | **Faye** | There are times when we feel like our medicines are missing. |
|  | **Anna** | When distributing medicines, we prioritize giving them to the indigent or the poorest due to the limited supplies. |
| ***IN:*** | *That’s a good strategy to prioritize given the limited supply.* | |
|  | ***Cath*** | *Some would even get mad at you.* |
| ***IN:*** | *Those are really the problem. But you need to be strict. Since you are just doing your job.* | |
|  | **Cath** | Sometimes, patients doubt the results of their BP. |
| ***IN:*** | I see. It’s like they’re doubting your credibility not knowing that you are trained especially with journal club and you are also guided with UTD, making you more reliable, and eventually earning their trust. Since you have already mentioned such challenges as the Internet, patient engagement. Other than those mentioned, is there anything else? | |
|  | **Faye** | For me, nothing will hinder me. If it is okay for everyone, then it is okay for me. |
|  | **Cath** | Sometimes, there are patients who are hard to convince to go to the center for consultation or if there are activities such as weighing children or checking pregnant women. |
|  | **Faye** | In our case, patients like those activities as it is a learning opportunity for them as well, especially for pregnant women. |
|  | **Anna** | It’s a different scenario in our barangay. Patients don’t like their children to be vaccinated. They don’t go to the center for check-ups either. |
|  | **Faye** | Some are scared and traumatized by the issue of dengvaxia. That's why you need to explain and make them understand well to convince them. Same with COVID vaccine. |
| ***IN*** | *There really is a lot of fake news and misinformation on that topic especially for those with no background in health education. This is where our roles as BHWs enter. To give proper education and enlightenment.* | |
|  | **Faye** | Yes, Mam. |
| ***IN*** | *You will really need a lot of patience.* | |
|  | **Faye** | Yes, a lot of patience indeed especially for those with different philosophies. |
| ***IN*** | *There really are different beliefs but we must be guided with the scientific side for the betterment of all. Now, I have a question. If you could change any of your current responsibilities, what would you change?* | |
|  | **All** | None |
|  | **Cath** | None as well, more on additional or improvement. |
|  | **Anna and Faye** | More on improvement as well. |
| ***IN*** | *Okay, so there is nothing you want to change but more for improvement. Such as?* | |
|  | **Dane** | Training and seminar. |
|  | **Anna** | We really need training, especially for us BNS who submit reports monthly or every January and July for profiling. |
| ***IN*** | Okay so for the next question, how do you feel about the use of technology or medical software for your scope of practice? | |
|  | **Cath** | It is alright especially in terms of communication as long as there is an available signal. |
| ***IN*** | *But are you comfortable using it such as UTD on your cell phone device or attending Zoom?* | |
|  | **All** | Yes. We’re comfortable. |
|  | **Dane** | If there is a signal. |
|  | **Cath** | It’s free so why not use it? |
|  | **Faye** | However, during the journal club, we feel shy about asking questions. |
| ***IN*** | *You can ask during the session, Ma’am. [Speaker] and the other doctors are very much willing to address it.* | |
|  | **Faye** | Yes, they are really willing to answer that’s why we really like the journal club. |
| ***IN*** | *At least, you were able to ask questions about the usual cases you encounter in the barangay. Do you think UTD is useful in your work?* | |
|  | **Faye** | Yes, whenever there are patients, I usually remember the learnings I read from UTD in handling cases. |
|  | **Anna** | Yes, I am able to share the knowledge with the patients so I can help them. |
| ***IN*** | *Moving on to the next question, what training opportunities for training or continuing professional development are available to you?* | |
|  | **Dane** | We attended a BLS training facilitated by the provincial health office. |
|  | **Faye** | We also attended Universal Health Care training by [speaker] from last year regarding the referral system. |
| ***IN*** | *What else?* | |
|  | **Cath** | Training on TB and Family Planning. |
| ***IN*** | *Are these trainings regular or one time?* | |
|  | **All** | One time only. |
|  | **Cath** | As for the TB training, there are said to be succeeding ones. |
|  | **All** | Yes, one time only. |
| ***IN*** | *So in one year, there really is no regular training except UTD journal club?* | |
|  | **Bea** | We have training on mental health awareness too and the one from [organization 1] and [organization 2]. That is twice a year, however, only focals from each barangay can attend. Only 10 barangays are covered by [organization 1]. To accommodate the others, they just went to the barangay, |
|  |  |  |
| ***IN*** | *So, [organization 1], [organization 2], and journal club are your usual training. Hopefully, you can regularly join the journal club,* | |
|  | **Cath** | Yes, Ma’am. My colleagues from [a certain barangay] reported the other day. |
| ***IN*** | Do you still regularly use UTD? | |
|  | **All** | Seldom. |
|  | **Bea** | In the barangay, we have our own phone for UTD, and we use that. |
|  | **Faye** | My phone is unable to install it. |
|  | **Bea** | We usually use telemedicine instead. |
|  | **Cath** | Yes, we presented about rubella and heat stroke which are very timely. |
| ***IN*** | *Did you try using UTD in handling patients?* | |
|  | **All** | Not yet, but the midwives in our barangay had. |
| ***IN*** | *I see. Were they able to coach you?* | |
|  | **All** | Yes, since there is a cellphone in barangay. |
| ***IN*** | What influenced you to use UTD? | |
|  | **Cath** | It is a good platform because of the comprehensive discussion, especially during the journal club where we can directly ask doc dans and he has a lot of insights to share. |
| ***IN*** | *It really is a huge help. How about the others?* | |
|  | **Dane** | It is nice since the topics for discussion are cases, we usually handle such as UTI, depression, etc. We are able to share that knowledge too to the patients. |
|  | **Cath** | Especially when we discussed rubella, it was very timely since a week after that, we conducted vaccination on rubella. We were able to easily explain to patients about the said virus and eventually convince them to get vaccinated. |
| ***IN*** | *That’s great, Mam. Actually, when the patients see that you know what you are saying, it would be easier for them to trust you.* | |
|  | **Cath** | That’s true. At first, they are hesitant since they are misinformed, especially about the dengvaxia case. We really had to explain to them well as to convince them, especially one family who had never had vaccinations before, but because we were able to enlighten them, they were finally convinced to get vaccinated. |
| ***IN*** | *UTD really is a big help, that’s why it’s good to have it downloaded on your devices. May I ask those who haven’t used UTD yet, what is the reason?* | |
|  | **Bea** | Cellphone, Ma’am. In our barangay, only one phone has UTD. It isn’t accessible to everyone. |
|  | **All** | We should have one for each. |
|  | **Dane** | Other phones are not compatible or with little memory. Not everyone has smartphones too. |
| ***IN*** | *If given the chance, what changes would you make to UpToDate or the journal clubs? How else can we improve it?* | |
|  | **Bea** | Perhaps, an offline version. |
| ***IN*** | *It has an offline version however it may consume a huge amount of your phone memory.* | |
|  | **Cath** | However, the offline version must still be updated from time to time. |
| ***IN*** | *Yes, every now and then, there are changes in the content. Now,*  *Would you recommend UTD use among primary care providers?* | |
|  | **All** | Yes |
| ***IN*** | *Why?* | |
|  | **Cath** | All the information you need is already there. |
|  | **Anna** | It is effective in a way that it aids us in explaining to patients or convincing them to seek consultation. |
|  | **Cath** | It is easy for you to explain to patients since you are guided. |
|  | **Dane** | is Easy to explain as well. |
| ***IN*** | *So would you convince your colleagues to use it?* | |
|  | **Faye** | Yes, Mam. It is a huge help. |
| ***IN*** | *If not only for the barriers right? How about you, Ma’am Emma?* | |
|  | **Emma** | It is nice since we are able to gain information about health. Especially since not everyone is a graduate or has a background in health. That is part of your continuous learning as long as you are willing. Is there anything else you wish to share about your role, the challenges you encounter at work, and your views on UpToDate and the journal clubs? |
|  |  |  |
|  | **Cath** | Are those that have been discussed in the journal club can be found in the UTD app? |
|  |  |  |
| ***IN*** | *Yes, Mam. All of it.* | |
|  | **Cath** | Okay, especially those cases usually encountered in barangay. It would be better too if there is a recorded video of the meeting to be uploaded for those who were not able to attend. |
| ***IN*** | *Yes, Ma’am. That’s actually a good question. Hopefully, all of us here can regularly attend the UTD journal club. If there are no more concerns, then this ends our discussion.*  *Again, thank you, everyone!* | |

**--[End Transcript (01:34:26)]—**
