# Supplementary material for: Resilience, ingenuity, and identity: A multi-level analysis of the Filipino community health worker experience in rural and remote municipalities in the Philippines
Source: PLOS Glob Public Health. 2025 Aug 18;5(8):e0004965. doi: 10.1371/journal.pgph.0004965 (PMC12360505; doi:10.1371/journal.pgph.0004965)
Supplement: S2 File — (ZIP) [file pgph.0004965.s003.zip › Rural PPCS Translation FGD 1.docx]

**Focus Group Discussion Translation**

Rural Site BHWs: Short Tenure

**Philippine Primary Care Studies**

NAST CHW Experience Study

**PRELIMINARY INFORMATION**

| Location: | Rural Health Unit of the rural site in Central Luzon |
| --- | --- |
| Date Recorded: | July 7, 2023 |
| Transcriber’s Remarks: | Participant names have been replaced with aliases to prevent identification. |
| List of Acronyms: | BNS = Barangay Nutrition Scholar  BHW = Barangay Health Worker  NDP = Nurse Deployment Program  UTD = UpToDate |

**TRANSCRIPTION**

**--[Begin Transcript (0:00:01)]--**

***IN:*** *How would you explain the work of a BHW to someone new in the community?*

**Jane:** BHWs disseminate information about health programs in the community. We also zero in topics related to nutrition. Health related information is focused on all people across the lifespan. We also take note of their health status and history.

***IN:*** *Do you focus on a particular subset of the population, or do you assess everyone?*

**Jane:** We focus on one topic across the lifespan. In my case, I disseminate nutrition related information for both old and young people.

***IN:*** *How bout you ma’am Cat?*

**Cat:** We encourage them to go to the health Center if they feel unwell. We explain that going to the health center would allow HCWs to thoroughly assess their vital signs.

***IN:*** *If some patients refuse to cooperate, is it part of your responsibility to encourage them?*

**Cat:** Yes, it is our job to encourage them. Their initial refusal should not stop us from repeatedly convincing them to avail of check-up.

***IN:*** *How about you Ma’am Tess?*

**Tess:** We must repeatedly remind them about vaccination schedules. Parents usually don’t have the initiative to go when they are not reminded.

***IN:*** *Does this mean that pedia cases involve interventions for both parent and child?*

**Cat:** Yes, we must really focus on the mothers. They are the hardest to convince.

**Tess:** It gets tough from time to time. Instead of focusing on the children alone, we work hard to make sure that mothers are agreeable to the plan.

***IN:*** *Having extended family at home is common in the Philippines. Do you also have to convince relatives?*

**Jane:** Yes, we do. The more we get to convince everyone, the more assurance we have that patients will adhere to the treatment plan.

***IN:*** *What strategy do you use to convince the extended family?*

**Tess:** The relatives are usually cooperative. However, the manner of convincing them will really depend on their personality. Our strategy will depend on their unique attitudes and beliefs.

***IN:*** *How about you Ma’am Gina?*

**Gina:** We are known as the professional Marites of our barangay. We know everything that happens in the community. We know who marries who. This helps us project the possibility of pregnancies in our barangay. Once a baby arrives, we encourage mothers to bring their children to the BHC. We are also the professional Marites of our barangay since we know who recently celebrated their birthdays and are ill.

**Tess:** We have a lot of information about everyone.

**Gina:** We thoroughly assess the family. We take the opportunity to provide medicine to fathers, when both parents are there, we take the opportunity to do family planning. We also take the opportunity ask if there are deaths in the family,

***IN:*** *How do you distribute government aid?*

**Gina:** We thoroughly assess if the person is there. We don’t include transient residents in the barangay. We only include family members who are residents in the barangay.

***IN:*** *Do you document this Ma’am Guba?*

**Gina:** Yes, we do, The barangay won’t release funds if we don’t have documentation.

***IN:*** *So you’re the Marites with screenshots?*

**Gina:** Yes, we do. But we’re proud when we accomplish the documentation for this. You just really must be patient if you want to get the answers you need from members of the community.

***IN:*** *That’s true. How about you ma’am Tine?*

**Tin:** The grind never stops. I’m assigned to an affluent barangay. Most people I screen have day jobs. This makes it difficult to talk to them during the day. When this happens, I usually go-back during off hours to get their cellphone number or Facebook account. I communicate most concerns through this platform. I also utilize online platforms when patients live in far-away areas.

**Gina:** I do this too. Having social media platforms allows me to remind parents to bring their children during vaccination schedules.

***IN:*** *Apart from what you mentioned, do you have other responsibilities to the barangay?*

**Jane:** We are the first ones to approach about any health-related concerns. I was a BNS prior to becoming a BHW. Before then, I was only assigned to educate patients about having a healthy diet and assisting them with anthropometric measurements. Now, I cover other health related programs as well.

***IN:*** *Apart from this, do you have other responsibilities to the barangay?*

**Tin:** We’re also assigned with clean-up drives.

***IN:*** *Can you elaborate more about your roles in this initiative?*

**Tin:** Everybody who works in the barangay is required to participate in community clean-up drives.

***IN:*** *Does your participation in clean-up drives help the community?*

**All:** Yes, it does.

***IN:*** *How do you say so?*

**Cat:** We are able to keep the surroundings clean.

**Jane:** The surroundings need to be clean since dirty localities can increase the number of community-acquired infections.

**All:** We also partake in our community’s disaster response.

***IN:*** *What’s your role during disasters,*

**Tess:** We direct people to their designated evacuation centers. We also help distribute food when there are evacuees in schools.

***IN:*** *Do you also partake in health assessments in evacuation centers?*

**Tess:** Yes, it’s one of our tie-ups with DSWD. We have to document any health related concerns during a disaster.

**Cat:** We do this since the barangay asks for documentation from us.

**Jane:** We must take note of these. BHWs are the first ones to be approached during emergencies in the evacuation center.

**Cat:** They usually don’t hesitate to approach us. They already trust us because of our previous encounters.

***IN:*** *Apart from what you mentioned, are there activities that BHWs spearhead?*

**Jane:** We spearhead some activities during nutrition month.

***IN:*** *What are some of the activities?*

**Jane:** We organize the “mothers’ class” and feeding programs in our barangay.

***IN:*** *Can you tell me more about the mothers’ class?*

**Jane:** These classes involve topics about promoting and maintaining the health of children in the barangay.

**All:** We also have family planning sessions and Zumba classes.

***IN:*** *During these activities, do you organize everything?*

**All:** Yes, we do.

**Gina:** We submit the necessary documentation for approval. However, we take care of all the groundwork.

***IN:*** *Are there other activities where BHWs take charge?*

**Cat:** We receive a stipend from the barangay during our birthday. When this happens, we usually celebrate with the community. We eat and celebrate with our patients.

***IN:*** *How do you feel about doing these activities during your birthday?*

**Cat:** We feel good about it. It’s also a good thing when barangay officials get to attend these celebrations. They can see the actual situation in the barangay. These celebrations also allow me to appreciate the simple joys in life.

**Cat:** Being a BHW trains you to become more empathetic. You become aware of how to approach people even when they don’t immediately tell you what they feel.

**Tin:** Through it all however, you need to have a lot of patience. Not all people are approachable or willing to cooperate. Regardless of this, you have to show them utmost compassion.

***IN:*** *What motivated you to become a BHW?*

**Jane:** I’m a nursing graduate. I applied as a BHW to refresh the medical knowledge that I have. I’m very happy about my current job. I’m able to use my learnings to serve the people.

***IN:*** *How about you Ma’am Tess?*

**Tess:** Someone encouraged me to try it out. However, I stayed because the job is very fulfilling.

**Gina:** I tried it out to pass time. My friend backed out when she was informed that no salary will be provided. I still pushed through so I can do something. Being a BHW also allows me to help my community. I also learn a lot of health-related skills. This includes learning how to assess a patient’s bp.

**Tin:** You really learn a lot of health-related skills. This includes health-history taking and assessment.

***IN:*** *You’ve mentioned that you learn a lot from being BHWs. Where else are you able to apply these skills?*

**Cat:** I wanted to work when my children grew up. I had no idea what being a BHW entailed. However, I wanted to try something new for the experience. Eventually, I learned that BHWs were not provided any salary. I continued because it felt fulfilling. You meet and learn to talk to a lot of people. You learn technical health related skills. However, you also learn how to relate to others. You also learn how to thoroughly document important information.

**Jane:** Yes, that’s true.

**Cat:** Remaining in the house is too boring. Being in the field allows me to socialize a lot. You look forward to hearing the stories of people.

**Tin:** I started with a 400 pesos allowance. I still continued on. Now, I earn 1,600 per month.

**Tess:** I was already working under the solid waste team prior to becoming a BHW. The captain kept trying to convince me, but I wasn’t sure if I could do it. Eventually however, I realized that being in the barangay would allow me to navigate the process more easily. I did everything I could to learn as much as I could. I would buy my own BP app and familiarize myself with it. I would also ask the guidance of other BHWs.

***IN:*** *How about you Ma’am Tin? How did you build your skillset?*

**Tin:** You need to have willingness. Our nurse and midwife helped a lot in building our skillset. Sadly, some BHWs and other higher-ups, would tell me that I couldn’t do it. I get discouraged from time to time. However, I tell myself that this is part of the job,

**All:** You just go on.

**Tin:** I get uncomfortable when someone snoops in my area. They should mind their own assignments. When things get too jarring, I just tell myself to mind my own business.

**Cat:** It really gets uncomfortable when people mind our area.

**Jane:** Yes, it is.

**Cat:** It’s best to stick to your area.

**Tin:** I don’t know their true intentions. They may do it out of the kindness of their hearts. However, it can really get uncomfortable when they mind your area.

***IN:*** *How does it feel when other people mind your area?*

**Cat:** I feel like they’re bypassing us. They should speak with us directly if they have concerns about our area. It feels like they’re using the superiority card to bypass us.

***IN:*** *What benefits do you get from being a BHW?*

**Gina:** We get goods during Christmas.

**Jane:** We are given grocery packages and stipends during Christmas.

***IN:*** *Do you also have access to free check-ups?*

**Tess:** Yes, we do.

**Tin:** We have access to this.

**Jane:** It depends. We’re privileged enough to have access to the Universal Health pilot program. Others who don’t know about it don’t get to access this benefit.

**Gina:** Are we covered by PhilHealth?

***IN:*** *I’m not certain.*

**Jane:** Some of us here are sponsored for a year.

***IN:*** *Is this program sponsored by PhilHealth?*

**Jane:** No, it’s a specific allotment for BHWs.

**Gina:** I hope we have a consistent Phil health provision.

***IN:*** *To clarify then, do you receive anything else apart from grocery packages?*

**All:**... We also receive ayuda.

***IN:*** *When do you receive this?*

**Jane:** We receive ayuda every month during the pandemic. After the surge, we only receive ayuda during Christmas.

**Tess:** We also receive some other monetary compensation.

**All:**.. We have an allowance.

**Jane:** I receive 430 pesos.

**Tin:** The 430 incentive comes from the municipal hall.

***IN:*** *Is this the same as the 1600 allotment you’ve mentioned prior?*

**Tin:** No, the 1600 allotment is provided by the barangay.

**Jane:** This is our honorarium.

**Tin:** The mayor also provides us 2000 every 3 months.

***IN:*** *Are you given the liberty to choose how the 1600 and 2000 will be spent?*

**All:** Yes.

**Tin:** Yes, but this is not enough especially because a lot of BHWs also must worry about their children’s school expenses.

**Tin:** It is what it is. We must make do of what is provided to us.

**Gina:** Maybe we’ll win the raffle someday. We just budget what we receive right now.

***IN:*** *What do you feel is the most touching part of your job?*

**Jane:** The kids. They treat you like their ate. Whenever we pass in the community, they always remember us people from the BHC who really help them out?

***IN:*** *Do you want to share anything else?*

**Tin:** It’s heartwarming when they say thank you. The simplest act of giving their medicines would make them so grateful. They tell us that they won’t get their medication without us.

**Tess:** This is true ma’am. We do house-to-house visits to distribute the medication to the residents.

**Jane:** Yes, we do.

**Gina:** We’re never strangers to them. They also say hi when we see each other in SM.

**Jane:** This is how it works.

**Cat:** Some seniors would also provide us snacks.

**Cat:** Some would give us bread.

***IN:*** *Does it give off the impression that you’re part of their family?*

**Jane:** Yes, it does.

**Tess:** Some would even message us to go to their house during special occasions.

**IN:** *Is this an extension of their gratitude?*

**All:** It feels so.

***IN:*** *Now, I’d like to ask if anyone guides and/or supervises your tasks?*

**Gina:** The midwife supervises us.

**Cat:** The barangay captain also provides us with orders.

***IN:*** *What’s the difference between directives provided by the barangay and directives provided by the midwife?*

**Tess:** The midwife focuses on health-related directives. The captain tends to give orders about anything under the sun. The captain tends to give emphasis on data collection though.

**IN:** Does this encompass health-related data?

**Tess:** This would help.

**Jane:** No, anything health related is mostly instructed by the midwife.

**All:** ... Yes, these orders mostly come from the midwife.

***IN:*** *Do orders come from anyone else apart from those whom you mentioned?*

**Jane:** Some health-related directives come from the RHU. However, the NDP and midwife take charge of providing specific instructions to us. We also coordinate with the leaders of the [organization]

***IN:*** *What does the [organization] do?*

**Jane:** They provide guidance through standardizing protocols.

***IN:*** *How does that work?*

**Gina:** Upon receipt of instructions from the midwife, the federation standardizes and/or looks into how BHWs will be involved in newly implemented health programs.

***IN:*** *How are directives brought down to you?*

**Gina:** It comes from the doctor.

**All:**...in the RHU.

**Gina:** The federation really looks into the roles and responsibilities of BHWs for a specific project. In sum, we are really the last ones to receive word about a specific project. Many consultations happen before our tasks get finalized.

***IN:*** *Can you give specific examples of responsibilities assigned by the midwife?*

**Gina:** For example, the midwife is given specific instructions about the [international infectious diseases program]. The midwife will then tell us specific instructions about the specific patient. They tell us to be discreet in handling them once they’ve been identified.

**Jane:** Yes, it’s confidential.

**Gina:** The MHO identifies which people need meds. Only the midwife knows who will receive it. The BHW knows whom they’re assigned to. The midwife is not always around. Hence, BHWs also take charge of following up their assigned patient’s health status. Blood donation projects relayed to the midwife are also endorsed to the BHW federation. BHWs would then recruit people from the community.

**Jane:** Immunization orders also come from the midwife. BHWs take charge of reminding people of their schedule.

**Tin:** We also provide the list of kids in our assigned areas.

***IN:*** *In essence, all orders are relayed through the midwife?*

**Tin:** Yes, they are. These health orders always come from the midwife.

***IN:*** *May I clarify the BHWs’ involvement in blood donation drives?*

**Cat:** The midwife would instruct us to recruit a certain number of people for the blood drive. We do house-to-house visits to check willing and/or qualified participants.

***IN:*** *To clarify, you really record the necessary data in advance?*

**Tess:** We record as much as we can including blood type. This helps us anticipate how much we’ll get.

***IN:*** *Are you given advice on how to execute tasks or do you figure out things on your own?*

**Jane:** We usually figure out things on our own. They just give us basic instructions on what to do for the patients.

***IN:*** *In essence, you personalize communication strategies with your patients?*

**Tin:** Yes, this is important to connect with them.

**Gina:** Yes, always personalize conversations and remain calm. People tend to post arguments on Facebook.

***IN:*** *Is the process of endorsing tasks clear or would you like to improve anything?*

**Jane:** We find it overwhelming from time to time especially when we receive orders about multiple health programs. We find it more confusing when we must execute orders from the barangay. I hope they give it one at a time.

**Cat:** I hope we are given more time to execute our tasks.

**All:** Yes, we hope the time frame can be adjusted.

**Jane:** They need everything stat.

**Tess:** Everyone needs everything asap.

**Jane:** They always tell us to submit everything asap.

**Tess:** This is the part that gets most distressing for us.

**Gina:** We are only given four days to accomplish something that takes longer.

**Jane:** We tend to accomplish less house-to-house related tasks during rushed orders. This happens because everyone needs to help out in accomp;shing things immediately.

***IN:*** *How do you manage documentation related tasks when everything needs to be accomplished asap?*

**Cat:** It would be helpful to have a laptop with us. Documentation can get really challenging especially when we must look at a lot of documents. A lot of new patients get logged into the system. Hence, a laptop would be helpful. I think we have one. However, things will be more readily accomplished if a laptop is available. Data has a higher likelihood of being wrongly encoded when only one person does it. When we’re not confident about the encoding, we often result in manual recording.

**Jane:** That’s true. There are times when we result in stacking folders. We’re not confident about encoded data.

**Cat:** There are really a lot of folders to sort through.

**Tin:** I do this as well. I manually count the demographic data of patients assigned to me.

**Jane:** In all honesty, manual encoding can get so confusing. It can get frustrating since we have

to pass these within the day.

**Tess:** I agree, in some cases we have to categorize files according to the work designations of our patients.

***IN:*** *Apart from documentation and validation, do you want to improve other areas of your job?*

**Tin:** It would be helpful if routine vaccinations were more systemic. It would be helpful to really predict who’ll go on what days.

**Jane:** Adarna

**Tin:** It would be helpful to train more mothers to read books to their children. It would also help to pay more attention to nutrition programs since this area lacks funding. A lot of children in my barangay are underweight.

**Jane:** We also hope to have more training and seminars.

**Tin:** Having more trainings and seminars would give us better knowledge on how to take care of kids.

**Jane:** Receiving more trainings and seminars would help us pass on knowledge to more BHWs.

**Tin:** Yes.

***IN:*** *How would these trainings be impactful?*

**Gina:** Trainings would be impactful if they focus on its pathos. We also have to work double time on passing down information to more people.

**Tin:** Making trainings emotionally impactful could lessen the tendency of people to make excuses.

**Gina:** This will make them realize how important it is to take care of themselves and their families.

***IN:*** *What aspect of your job would you like to change?*

**Jane:** I hope they give less orders.

**All:**..We agree.

**Jane:** As a former BNS worker, I could compare the difference between both jobs. I do more as a BHW. There are times when I have to validate community-level data while also attending to my tasks in the health center.

***IN:*** *How will you improve this if given the opportunity to decide?*

**Jane:** I hope they can hire more workers.

**Gina:** I hope we get assigned to a smaller number of patients. From what I know, each BHW should handle 300-500 patients only.

**Mary**: In theory, my barangay mandates that each BHW only handle 10 households. This is not followed.

**Gina:** I’m in-charge of households.

**Tin:** I take charge of 109. This is a lot.

**Cat:** I’m in-charge of 260.

**Jane:** I’m in charge of 500.

**Tess:** I take care of 654 households. My hands hurt from documenting for a lot of people.

**Cat:** That’s a lot. In my case, I have to handle two areas.

***IN:*** *Apart from manpower concerns, do you wish to change anything else?*

**Gina:** If we ask for more demands, we might get fired.

**Jane:** I’m also afraid of that.

**Cat:** I get the impression that I will get fired if I complain about anything.

**Tin:** This is also why a lot of people don’t apply. There are also salary issues to consider. People don’t apply since being a BHW is purely voluntary in nature.

**Cat:** My sister would always tell me that we’re heroes.

***IN:*** *How about improvement wise?*

**Cat:** I hope they recognize that we really do a lot. Some officials have the impression that we only gossip in the BHC.

**Gina:** I think I should be more internal. The system won’t change anytime soon. It’s better to adjust my schedule so I could do more documentation despite the limited time I’m given.

***IN:*** *A lot of you mentioned documentation concerns. How would the introduction of technology impact these tasks?*

**Tess:** Having gadgets would help us accomplish a lot. However, we should also have trainings on how to use them.

**Gina:** There should be a lot of trainings. This will ensure that we use the gadgets provided to us.

**Tess:** Excel training would be useful. This will speed up the encoding process.

**Tin:** I just worry that I’d take too much time familiarizing myself with the gadget instead of accomplishing a task.

**Jane:** Me too, I’ll take a lot of time learning how to navigate the application.

***IN:*** *If you take a lot of time learning gadgets, how would that impact actual utilization?*

**Gina:** I would prefer manually writing down information instead.

**Tin:** Me too. We’re already used to this anyway.

**Cat:** Maybe they would hire another person who’ll just focus on encoding.

**All:**..Yes, that would be efficient.

**Cat:** Yes, hiring someone would be most optimal. This means that BHWs don’t have to spend hours in training. We can focus on doing more groundwork.

**Tin:** However, I do acknowledge that putting in the time to learn would help us navigate the system.

**Gina:** That’s true. Although this is too time consuming for now.

**Tess:** I tried learning this before by asking help from the NDP. I was able to encode some data. However, it was too much of a hassle to correct data that I wrongly encoded.

**Cat:** I was able to try encoding before. However, I wasn't able to maximize a lot of features.

**Tess:** Maybe simple or basic trainings will do.

**Jane:** Anyway, these trainings will only be effective if we have the willingness. To learn.

***IN:*** *Is there a way to improve willingness?*

**Jane:** I think willingness to learn will really come from within. There are people who quit the job when they already find it difficult to learn new skills. The laptop is always available if we want to use it. Both our NDP and midwife are also willing to teach. However, some BHWs really refuse to learn. They feel like all files will be deleted if they touch the computer.

**Gina:** This is why a lot of people don’t like to use it.

**Tess:** I’m okay with learning it.

***IN:*** *If people don’t like using gadgets, how do they record patient data?*

**Tin:** We just write everything down.

**Gina:** We create a manual profile for all patients.

**Tess:** Everything is logged down in our notebook. We really record the programs where each resident partook in.

**Tin:** We record the medications each senior citizen receives. We also record how many COVID vaccines have been availed by a certain patient.

***IN:*** *What happens when there is a shortage of materials in the center?*

**Jane:** It happens. Materials get misplaced or lost from time to time.

***IN:*** *Can you expound on that?*

**Jane:** The health supplies we used during the pandemic were never returned to us. The aftermath of this was very hard. When we must monitor potential maternal HTN cases, we can’t use anything. When this happens, we request materials from higher-ups.

***IN:*** *Does this request materialize?*

**Jane:** Sometimes it doesn’t. In our barangay, we regularly receive the supplies we need. However, we still experience a lot of shortage. Residents would not return the nebulizers they borrowed.

**Tess:** This is why we really must note everything.

**Jane:** Even when we note everything, there are people who don’t return materials.

**Gina:** They always reason out that they’ll return it anyway. So why rush?

***IN:*** *When this happens, do you still try to get it?*

**All: ...** Yes, we do.

**Jane:** We disinfect all materials once they return it.

***IN:*** *Do the patients usually agree with this?*

**Jane:** Yes, they do. It just takes a long time for them to return it.

***IN:*** *How do your conversations usually go?*

**Jane:** Difficult to say the least. They would always tell me that they’ll return everything in due time. They can’t return it immediately since their neighbor still borrowed it.

***IN:*** *So they lend it to others?*

**All**:..Yes.

**JANE:** Yes, they do. Our materials go missing in action from time to time.

**Tin:** Whenever a patient tells us that they’ll return it to the center, I try to really go to their house and ask for it.

***IN:*** *How are other patients affected when there is a shortage of supplies?*

**Jane:** There is a delay in their health assessments. We can’t consistently monitor everyone who has hypertension concerns.

***IN:*** *How do you resolve this shortage?*

**Jane:** We borrow from BHWs who have their own things.

***IN:*** *Can someone from the upland barangay share their experience?*

**All:** ..all of us are from the lowland barangay.

***IN:*** *How do you handle patients who live in geographically challenging areas?*

**Jane:** We regularly experience floods in our area. There are delays in immunization during high tide. Having a cellphone allows us to message them about the delay. Unfortunately, residents also refuse to go to the center. They tell us that it’s hard to travel during the high tide.

***IN:*** *Apart from that, are there other calamities that make BHW work challenging?*

**Tess:** There are a lot of poor residents in Pugad Lawin. This locality is situated far from the BHC. Residents don’t maximize primary care service since they find transportation fare too expensive. We usually do check-ups in the nearby chapel to resolve this issue. We also hold immunizations there.

***IN:*** *It became a health center?*

**Tess:** Yes, it did.

**Cat:** I have another problem in my area?

***IN:*** *Can you expound on this?*

**Cat:** I’m assigned to an affluent community. They usually refuse to entertain me. People don’t allow me to do health profiles or take their bp.

**Tin:** It’s just ironic. These people are quick to entertain us when the barangay will provide a stipend.

***IN:*** *Are there any problems in your barangay that you’d like to open Ma’am Tin?*

**Tin:** People are usually cooperative. It just gets extra challenging to do area work during high tide or typhoons.

***IN:*** *Let’s transition to UTD utilization. Has everyone used UTD?*

**All:** …Yes, we have.

***IN:*** *How often have you used UTD outside of journal clubs?*

**Jane:** We usually ask help from our midwife to search information in UTD. We channel all concerns through our midwife.

**Gina:** I find it hard to use UTD at home. I don’t have Wi-Fi. I only get to access it during our sessions in the BHC.

***IN:*** *To clarify, is someone able to use UTD outside sessions?*

**Gina:** It’s hard to access UTD. I try to buy load for it. This is not enough. I usually don’t have signal in our area.

**Jane:** The required capacity for UTD is too big. Our smartphones are not sophisticated enough for the application’s specifications.

**Tin:** I don’t use it on my own. I just use it when I have the time to access it with other BHWs in the center.

***IN:*** *What do you usually search while using this app together?*

**Gina:** I have difficulty with this. The app closes whenever I use it.

**Tin:** I’m not able to use it. I’m unable to participate in these sessions.

**Jane:** We have an available phone with UTD installed in it. However, the midwife has it.

***IN:*** *So only the midwife has access to it?*

**Jane:** Yes. We usually pay attention during sessions.

**Gina:** Yes, we mostly utilize it during sessions.

**Jane:** Yes, we usually search topics as a team.

***IN:*** *How about you ma’am, what’s your experience with the application?*

**Cat:** I downloaded the application. However, I only use it during sessions.

**Tin:** Same, I have the app downloaded on my phone. I only use it during sessions though.

**Cat:** I use it to read about commonly occurring diseases in our community. I try to read-up on commonly occurring signs of certain diseases before assessing patients. This is important since midwives usually ask us about individuals we must take extra care of. The application also helps us gain knowledge on what advice to give residents. It gives us direction on what they can and cannot eat. UTD allows us to give accurate answers.

**Jane:** We are more likely to give out health education based on facts.

**Cat:** We may not be able to diagnose, but UTD helps us impart useful knowledge to our patients.

***IN:*** *What gadgets do you use to access UTD?*

**All**: ..We use our phones.

***IN:*** *Ma’am Tess, have you used UTD outside of journal clubs?*

**Tess:** No, I haven’t. I’M only able to use it when I’m on duty with the midwife.

***IN:*** *What do you search for on UTD?*

**Tess:** Someone help us with this.

**Jane:** That person assists us during sessions.

**Tess:** We are usually provided a link and instructions to access UTD.

***IN:*** *Do you try it on your own?*

**Tess:** I do. However, I get so lost while using the application. I’m also too busy to explore it as frequently as possible.

**Jane:** We also experience some problems with our accounts. We forget the password we used to open it.

***IN:*** *What other factors hinder UTD use?*

**Jane:** It takes time to come up with a plan of action. We use our own knowledge along with input from our midwife. This helps us respond to patients as quickly as we should.

**Gina:** We also find it hard to follow…

***IN:*** *Follow what exactly, ma’am?*

**Jane:** The step-by-step instructions on how to use it.

**Gina:** I get so confused while navigating the application.

**Cat:** I find the text in English difficult to understand. It gets too overwhelming. I ask the midwife instead.

**Jane:** This is our mentality with UTD usage.

**Cat:** This is why UTD sessions are so helpful. We understand lectures in Filipino.

**Tess:** We try the translate feature. However, this is often inaccurate.

**Cat:** Isn’t it possible to request a Filipino version of the application?

***IN:*** *This feature is not yet available.*

**Cat:** BHWs will only use the app if it’s in a language that they can understand.

**Jane:** It’s also difficult to comprehend medical terms.

***IN:*** *How do you feel about the length of text?*

**Cat:** It’s too long. The text gets overwhelming to read.

**Tess:** It becomes even more difficult when you have poor eyesight.

**Jane:** It’s not straight to the point.

**Tess:** I tend to forget a lot of what I’ve read since there’s a lot to take in.

***IN:*** *Would the integration of visuals help?*

**Cat:** Yes, it would. Pictures would help us visualize the information that we need. Pictures would help us understand information in layman’s terms.

**Jane:** It’s too wordy.

**Cat:** You must put in so much effort to digest the information.

***IN:*** *Do you find it challenging to visualize the concepts?*

**Tess:** Yes, we do. Having everything in English makes it more challenging to understand the information posted.

**Jane:** It only shows pictures from books. We want to see images of how it manifests on a person.

**Cat:** It really needs to be simplified.

**Jane:** I agree.

**Tess:** It would really help to have it in Filipino.

***IN:*** *How would you feel if UTD’s features will be improved?*

**Jane:** I think I will. It’s difficult to be ultra-dependent on the midwife. Knowing how to use UTD will make us more independent in our practice.

**Cat:** We’ll also be more confident in handling patients. There is less doubt that we’re giving the right treatment since information came from UTD.

**Jane:** It gives us a scientific basis for what we do.

***IN:*** *How about you, ma’am?*

**Gina:** I will use it more if previously mentioned issues are resolved.

***IN:*** *Why so?*

**Gina:** It helps me care for patients more confidently without being dependent on the midwife.

***IN:*** *How do you feel about journal clubs?*

**Tess:** I really want to learn from the sessions. I understand that learning from them would help me take care of the community and my family even more. However, I only get to attend these clubs when I’m at the BHC. I find it difficult to attend sessions while I’m at home. I don’t have a device that can support online meetings.

***IN:*** *Are you able to attend Ma’am Jane?*

**Jane:** Yes, I am.

***IN:*** *What are your thoughts on the session?*

**Jane:** I find them useful. I can take a screenshot of the usual markers and apply this information while assessing patients. This helps me anticipate the needs of patients who come to the BHC.

***IN:*** *How does this apply to your everyday practice?*

**Jane:** I’m able to explain why patients feel the way they do. I’m able to guide them more.

**Cat:** It’s very useful when I must look for quick remedies to various ailments. Herbal medicines can help in curing people. However, these usually take time before the desired effect is achieved. UTD gives me more options that will help my patient regain optimal health.

***IN:*** *Would you like to improve anything in the journal clubs?*

**Tin:** I can’t think of any.

***IN:*** *How about you ma’am?*

**Jane:** I appreciate that sessions are not lengthy. The open forums also help me clarify information I don’t understand.

**Cat:** I hope we get to monitor the progress of our patient. We provide remedies based on what we’ve read from UTD. However, we can only be certain that our treatment plan was effective if we see it’s long term outcome on the patient?

***IN:*** *Would you recommend UTD to other PCPs?*

**Jane:** Yes, I will. This will make us less dependent on midwives. This will keep our knowledge updated.

***IN:*** *Would you also recommend it to PCPs other than BHWs?*

**Jane:** Yes, but not so much. Doctors and midwives already know what they’re doing.

**Gina:** I agree. Midwives are already well versed about different ailments.

**Cat:** I think joint sessions with the midwife will help. This will also help us brainstorm what to do when we encounter a patient with a certain disease.

**Cat:** I’d mostly recommend it to BHWs. They can immediately apply what they learn to their families.

***IN:*** *Would you recommend it to other HCWs ma’am Tess?*

**Tess:** I would recommend it to those who are not familiar with it yet. This will help us provide emergency first aid.

**Cat:** Prevention is better than cure. This will help us manage a patient’s symptoms before it gets worse.

***IN:*** *How can we encourage more people to use UTD?*

**Tess:** I must believe in its capacity first. I know that it can help me take better care of both my family and patients. However, I haven’t used it as much to see that this benefit is possible for me. I promise to explore the app more. Once I’m fully convinced, I’m confident that will encourage more people to use UTD.

***IN:*** *How about you ma’am Gina?*

**Gina:** I agree with her sentiments.

**Jane:** I would emphasize that UTD is helpful in providing emergency first aid. I’ll tell them that it would be difficult especially when they must buy their own load or look for a signal to access the application. However, it would be very useful in managing the conditions of our future patients.

***IN:*** *What can we do to solidify people’s confidence?*

**Jane:** More trainings.

**Cat:** I fully agree with this. The application won’t be utilized if people are not familiar with navigating it.

**--[End Transcript (1:22:08)]--**
