# Supplementary material for: Resilience, ingenuity, and identity: A multi-level analysis of the Filipino community health worker experience in rural and remote municipalities in the Philippines
Source: PLOS Glob Public Health. 2025 Aug 18;5(8):e0004965. doi: 10.1371/journal.pgph.0004965 (PMC12360505; doi:10.1371/journal.pgph.0004965)
Supplement: S2 File — (ZIP) [file pgph.0004965.s003.zip › 2022-02-17 PPCS Translation FGD 3 .docx]

**Focus Group Discussion Transcription**

Rural Site BHWs: Long Tenure

**Philippine Primary Care Studies**

NAST CHW Experience Study

**PRELIMINARY INFORMATION**

| Location: | Rural Health Unit of the rural site in Central Luzon |
| --- | --- |
| Date Recorded: | July 7, 2023 |
| Transcriber’s Remarks: | Participant names have been replaced with aliases to prevent identification. |
| List of Acronyms: | NDP = Nurse Deployment Program  BHW = Barangay Health Worker  UTD = UpToDate |

**TRANSLATION**

**--[Begin Transcript (0:00:01)]--**

| ***IN:*** | *How would you describe your job to someone who has recently arrived in your locality?* | |
| --- | --- | --- |
|  | **Tanya:** | I first tell them that I’m the BHW introduced in this area. I ask them if they’re living with any pregnant person. If this is the case, I encourage them to avail of check-ups in the BHC. They should not miss this opportunity since doctors are not in the BHC everyday. I do this since it’s my job to bring health programs to the residents of the barangay. Approaching people also allows us to correct erroneous health beliefs. For example, we stress the importance of regular prenatal visits. I don’t want residents to do home births simply because they had no other choice. Having regular health check-ups allows us to build a health plan for the mother and the baby. It also allows us to have a clear inventory of births in the barangay. |
| ***IN:*** | *How about you Ma’am Lina?* | |
|  | **Lina:** | BHWs work hand in hand with midwives and NDPs to execute health related programs. We are known as the professional Marites of our barangay. We do house-to-house visits to let residents know what health programs they may avail of. This includes informing them about available vaccines. We also do an inventory of animals in our barangay. |
| ***IN:*** | *Why is it important to have a census of animals in your barangay?* | |
|  | **Lina:** | This helps us project what vaccines may be needed in the future. |
| ***IN:*** | *How about you Ma’am Belinda?* | |
|  | **Belinda:** | We help residents understand who to look for when they have specific health concerns. |
| ***IN:*** | *Can you tell us more about this?* | |
|  | **Belinda:** | Some patients are too shy to immediately open up their concerns in the BHC. We convince them that it’s okay to go to your check-up. We are here to support you. |
| ***IN:*** | *How about you Ma’am Gina?* | |
|  | **Gina:** | As a BHW, I create family profiles. I record how many people are pregnant and their length of stay in the barangay. I also got their cellphone number. This helps me update them about check-ups in the BHC. It is also important for me to do a profiling of animals in the barangay. This helps patients identify who to talk to in case a relative gets injured. This also helps us estimate what vaccines are needed in the future. |
| ***IN:*** | *How about you Ma’am Karen?* | |
|  | **Karen:** | I’m a professional Marites. If someone recently relocated, I ask them how long they’ll be staying in the barangay. I ask them if they have a child aged 0-59 months. I also ask them if they have a teenage daughter with them. This helps me identify who is potentially at risk for teenage pregnancy. As mentioned by my colleagues, the number of people in the household also helps me estimate how many vaccines are needed.  When I notice people who recently gained weight, I initiate friendly conversation with them. I use this as a starting point to ask if they have any recent health concerns they would like to share. I also approach pregnant teenagers to ask about any assistance they might need. |
| ***IN:*** | *You mentioned a big difference between the responsibilities of BHWs then and now. Can you elaborate more about this?* | |
|  | **Tanya:** | Training was very informal before. It would happen under a tree or wherever space was available. Now, everything is more formal and standardized. There is also a budget for training.  Having cellular phones would also allow us to screenshot discussions. This would help us read about topics during our free time. Through gadgets, we are also able to pass on information to colleagues that did not attend the training. Cell Phones also help us contact patients who are missing in action. It also helps us immediately record patient data. |
| ***IN:*** | *How about you Nanay Belinda, what differences have you observed between the workload of BHWs then and now?* | |
|  | **Belinda:** | Documentation is more efficient nowadays. We are able to do more work since everything is simply encoded in a computer. Having more free time gives us additional legroom to read UTD. |
| ***IN:*** | *Are there particular activities where BHWs take charge?* | |
|  | **Gina:** | As an officer, I have team building activities lined-up for my colleagues. These activities will help us build camaraderie and character. We have executed some of these activities. We have recently conducted a leadership training seminar for officers of our federation.  This event developed the skills of my colleagues. The training helped them interact and communicate more smoothly with their fellow BHWs. They also learned effective strategies to survive a challenging activity. We realized that we are able to apply these learnings to our BHW. We realized that no matter how difficult a challenge is, there would always be a way to survive it. |
| ***IN:*** | *Do you have community focused projects?* | |
|  | **Gina** | We are planning to organize bingo events or volleyball leagues. BHWs don’t receive regular compensation. These activities would help sustain the needs of BHWs. If a colleague of ours gets sick, we are able to contribute to their expenses. We could also use other proceeds to help the families of BHWs who recently passed on. |
|  | **Lina:** | We organize Zumba sessions in our barangay. This strengthens the bonding between community members. |
| ***IN:*** | *Moving forward, I would like to ask Ma’am Karen what encouraged you to become a BHW?* | |
|  | **Karen:** | My father provided birthing services back then. I would go with him to far flung barangays to help out with his job. Eventually, a friend encouraged me to apply as a BHW.  I did not receive any compensation back then. However, I continued serving my community because they needed me. The midwife in our barangay is more confident to go to unfamiliar places in our barangay as I am able to help her navigate it.  I feel very happy about my job until now. I may not receive any compensation, I feel fulfilled because I’m able to help people in my barangay. It feels so heartwarming when residents express their gratitude.  Sometimes, I feel scared since they might remove me because of old age. I would voluntarily attend check-ups to show that I’m capable of doing my job. |
| ***IN:*** | *Who else would like to share their journey about being BHWs/?* | |
|  | **Belinda:** | I entered the profession since my husband was mostly away. Being a BHW allowed me to do something more meaningful with my life. Regardless of how it started, I stayed in this profession because I love it. I feel fulfilled that I’m able to help my community. |
|  | **Tanya:** | I stayed because I enjoyed the bond we have as BHWs. During the height of the pandemic, I would do house-to-house visits to assist patients. The dangers of COVID-19 did not stop my colleagues from helping me out. They would always accompany me to provide care for the patient.  Many individuals also applied to become BHWs during the pandemic. People were encouraged to apply since they are given the responsibility to distribute monetary aid. I always told them that BHW work goes beyond this. Surprisingly, all of them stayed even after the pandemic. |
| ***IN:*** | *Now, I would like to ask if you receive any compensation or benefits?* | |
|  | **Lina:** | Some BHWs receive an allowance. BHWs in our barangay receive Php 1,200 monthly. Our mayor also gives a quarterly allowance. The provincial government also gives a quarterly allowance of Php 1,500. |
|  | **Tanya:** | BHWs from one barangay only received compensation when a new barangay captain was elected. During our time, only Php 30 pesos was split among barangay health workers. |
|  | **Gina:** | BHWs from one barangay don’t have a stable allowance. Three months would pass by before they receive a salary. In my case, I only received compensation after five years of being of service.  I have a bigger allowance now. However, my responsibilities also increased. I was only assigned to call patients about their vaccination schedules back then. Now, I’m also in-charge of listing down who needs to receive certain vaccines. During the next salary increase I was also given the responsibility of identifying individuals to be included in our [local reproductive health program]. I also have the responsibility of encouraging pregnant women to attend prenatal check-ups in the health center. Upon implementation of other ]primary care programs] it also became my responsibility to promote this to the residents of my barangay. My allowance also increased during COVID. |
|  | **Karen:** | Despite the salary increase, we were removed from our post when a new barangay captain got elected. Now, our compensation would also decrease if health programs are nor their priority. In these experiences, we showed them our dedication to work despite these uncertainties. We show them that we serve the community out of our dedication and kindness. Eventually, the barangay captain decided to give us what we were due.  One BHW was also removed because of political conflict. Sometimes they are reinstated in their post, sometimes they aren’t. Lina is one of those who experienced this. |
|  | **Lina:** | Yes, I did. |
|  | **Tanya:** | Having no allowance didn’t bother us back then. When we wanted to have feeding programs, we’d spend out of pocket. We also solicit from everybody we know to get the budget we need to help our community, |
| ***IN:*** | *Since you’ve mentioned collaborating with the midwife, I’d like to ask if anyone provides you guidance?* | |
|  | **Belinda:** | Many people do. Some of these include our Mayor and the RHU staff. We would feel lost without them. It would be difficult for us to help our community if they won’t guide us. |
|  | **Lina:** | Directives come from the midwife and the NDP. BHWs would also guide fellow BHWs. |
|  | **Tanya:** | There are times when midwives and BHWs don’t get along. Orders were more smoothly executed when everyone maintains a good working relationship. BHWs from different “factions” would start helping each other out when conflicts get resolved. |
| ***IN:*** | *Can you share some advice you receive from NDPs and midwives?* | |
|  | **Lina:** | They relay and give instructions about different health programs. They give us specific instructions on what the involvements of BHWs will be. The guiding hand of our barangay captain is also helpful in making the project happen. They would provide us transportation support when we have activities far-away. We haven’t encountered any inconvenience when we worked with them. As long as we provide supporting documents, our requests get granted. |
|  | **Tanya:** | Multiple vehicles are available when we need them for our projects. |
| ***IN:*** | *Are you given training on how to handle special patients?* | |
|  | **Lina:** | We have seminars on how to handle different kinds of patients. BHWs who attend the seminar are tasked with sharing their knowledge to colleagues who did not attend the seminar. During our monthly meetings, we also orient fellow BHWs on the correct approach when talking to the patient. NDPs would also announce seminars and future seminars to us. |
|  | **Belinda:** | Having our own “diskarte” is also important in executing orders. Some patients refuse to adhere to health programs. We get the opportunity to really know them. This helps us convince them to attend health programs. They also open-up more. |
| ***IN:*** | *What factors or resources can help you become more efficient as BHWs?* | |
|  | **Tanya:** | It is important to maintain a good working relationship with the barangay captain and the midwife. We should learn to set aside personal conflicts. If we don’t like their performance, we must learn how to approach them. A good working relationship goes a long way in helping our barangay thrive. |
|  | **Lina:** | It is always important to set-aside time for self-reflection. We may not like how people in the barangay treat us, we must remain humble to them. |
|  | **Karen:** | My job becomes easier when I get their cellphone number. I’m able to update them about the availability of medications. I don’t have to go to their houses. Having a cellphone also helps me monitor available resources. Through pictures, I can check if they have a CR or not. |
| ***IN:*** | *Having a cellphone helps a lot. May I know if the barangay provides a load allowance?* | |
|  | **Gina:** | We have learned to accept that most expenses will come out of pocket. New BHWs should learn to accept that the barangay cannot give everything they need.  You have to continuously engage with residents. This incentivizes them to be more proactive in taking their help. When you’re involved, neighbors will also help you convince their peers to avail of check-ups. Having a cellphone helps us show them that we are very involved in online and offline spaces. |
| ***IN:*** | *Which among your responsibilities would you like to change or improve?* | |
|  | **Karen:** | I would not like to change anything. I can see that all my efforts help maintain the health of my community. In fact, I’m willing to take on additional orders if need be. |
|  | **Tanya:** | I have no problem if my responsibilities will change or increase. We will be left behind if we remain unwilling to execute new orders that will improve the health of our community. Our BHC does not have Wi-Fi. I availed a Wi-Fi service so I could reach out to more people. |
| ***IN:*** | *What improvements would you like to implement?* | |
|  | **Lina:** | Proactively debunking health stigmas encourages more patients to go to health centers. Through our [locally lead program on infectious diseases], residents are more willing to go to the center to get their medicines and have their weight regularly assessed. I hope this happens for other government programs.  I hope our allowance will also increase. We have a lot of responsibilities nowadays. New applicants would also ask us about the compensation. They would reason out that they need it to sustain their families. Some BHWs I know resigned because they were not given enough compensation. Having an allowance would make BHWs more confident that they can support their own lifestyle.  Sadly, applicants nowadays see the BHW profession as a job. When the veterans retire, I’m afraid no one will volunteer. |
|  | **Tanya:** | Before I stepped down as federation president, I assigned one veteran to each barangay. New hires tend to follow veterans whether they receive compensation or not. |
| ***IN:*** | *Now, let’s discuss UTD. Has someone used the application outside the seminar?* | |
|  | **Belinda:** | No, we don’t have signal. |
|  | **Karen:** | No, I’m not familiar with how to use my phone. |
|  | **Gina:** | I am able to use UTD in my upland barangay. When a patient asks me about how to help their children, I use UTD to search for light remedies. I refrain from giving extensive health advice. This may have an untoward effect on the patient. |
|  | **Lina:** | I use UTD to help my brother when he’s sick. I translate the reading material to Tagalog. This helps me understand it more. UTD also helps me increase my knowledge. I have less difficulty comprehending medical terms.  However, my access is limited. I can only use it when I have Wi-Fi. |
|  | **Tanya:** | I used UTD to help my husband before he passed on. I was able to understand the progression of his disease. This helped me anticipate his needs. |
| ***IN:*** | *What gadget did you use to access UTD?* | |
|  | **All:** | Cellphone. |
| ***IN:*** | *What other factors encouraged you to use UTD?* | |
|  | **Lina:** | I love reading. UTD helped me manage the signs and symptoms of my diabetes. I also use it to understand drug reactions. I prefer UTD for online reading since I’m certain that my source is correct.  UTD also helps me provide better support to the midwife for the health needs of my patient. |
| ***IN:*** | *What challenges have you encountered from using UTD?* | |
|  | **Karen:** | I’m not familiar with using a cellphone. I only apply what I learn from the seminars to help my patients and my family. |
|  | **Belinda:** | I’m also not familiar with using a phone. I find navigating the application difficult. Searching up desired reading materials is also difficult since I don’t have regular Wi-Fi access. I can’t study as frequently as I want. |
| ***IN:*** | *What features of the application do you wish to improve?* | |
|  | **Lina:** | I find it straightforward. I’m able to find the specific information I need immediately. It was difficult for me to access after the subscription ended. I cannot access offline data since I forgot my password. |
|  | **Gina:** | I appreciate the voice search feature of UTD. I can use it to search up certain information about certain diseases. It’s not hard to read content from an application. I translate the content to Tagalog if I find it difficult to understand in English. |
|  | **Tanya:** | I find it easy to navigate. I translate everything in Filipino when I don’t understand the content. |
| ***IN:*** | *How do you find the journal clubs?* | |
|  | **Karen:** | I find the actual presentations helpful. Having specific case studies helps me understand the disease more. |
|  | **Tanya:** | The journal clubs are effective. They help me understand outdated health beliefs. I’m more confident about the health advice I give since they come from experts. Journal clubs also help us identify the differences between similar conditions like measles and smallpox. It would really help if we encourage more people to use it. |
|  | **Lina:** | I feel really knowledgeable after each session. Most of us did not have the opportunity to attend form studies. However, journal clubs give us invaluable knowledge to share with our patients. The lecturers are really effective. I remember what they teach. |
| ***IN:*** | *What can we improve about the journal clubs?* | |
|  | **Lina:** | I hope all BHWs have stable internet access during the session. We have difficulty understanding the concept when we keep on getting disconnected. |
|  | **Tanya:** | The seminars are okay. The manner of presenting information makes us feel like we’re in a face-to-face setting. The Q&A portion helps us clarify concepts that are difficult to comprehend. |
|  | **Lina:** | I hope more BHWs learn how to mute. It’s distracting when we hear them talk in the background. Time is also wasted when the speaker has to tell them to focus. |
| ***IN:*** | *Will you recommend UTD to other PCPs?* | |
|  | **Lina:** | Yes, it’s a big help to us. Our knowledge about diseases really increases. We become more confident in interacting with patients. |
|  | **Gina:** | We hope that the information in UTD reaches more patients. It’s also helpful when patients know about the different diseases they can encounter. |
|  | **Belinda:** | I appreciate UTD even if I don’t know how to use a phone. I can ask other colleagues about the topics they’ve read. UTD helps us pass on credible information to colleagues and patients. |
| ***IN:*** | *How can we encourage more BHWs to continuously use UTD?* | |
|  | **Gina:** | We are very willing to use the application. However, we also wish to receive additional guidance about the scope of our practice. We don’t want to mistakenly treat patients or provide something that only doctors are allowed to give. |
|  | **Karen:** | I don’t know how to encourage others since I don’t use the application myself. Despite this, I appreciate UTD since I’m able to get credible information from my colleagues.  I have to convince myself about being open to gadget use and applications before I can convince others. |
|  | **Lina:** | More BHWs will be encouraged if we have regular gatherings where we read information from the app. We could then share what we understood from reading the informational material. |
|  | **Gina:** | We hope additional gadgets will be provided. Not all BHWs have memory cards or cell phones that could accommodate a heavy duty application. |

**--[End Transcript (1:41:58)]--**
