# Supplementary material for: Resilience, ingenuity, and identity: A multi-level analysis of the Filipino community health worker experience in rural and remote municipalities in the Philippines
Source: PLOS Glob Public Health. 2025 Aug 18;5(8):e0004965. doi: 10.1371/journal.pgph.0004965 (PMC12360505; doi:10.1371/journal.pgph.0004965)
Supplement: S2 File — (ZIP) [file pgph.0004965.s003.zip › 2022-02-17 PPCS Translation FGD 2.docx]

**Focus Group Discussion Transcription**

Mid Tenure: Rural BHWs

**Philippine Primary Care Studies**

NAST CHW Experience Study

**PRELIMINARY INFORMATION**

| Location: | Rural Health Unit of the rural site in Central Luzon |  |
| --- | --- | --- |
| Date Recorded: | July 7, 2023 |  |
| Transcriber’s Remarks: | Participant names have been replaced with aliases to prevent identification. |  |
| List of Acronyms: | NDP = Nurse Deployment Program  BHW = Barangay Health Worker  UTD = UpToDate |  |

**TRANSLATION**

**--[Begin Transcript (insert time)]—**

| ***IN:*** | *How would you describe the roles and responsibilities of BHWs to newcomers in your barangay?* | |
| --- | --- | --- |
|  | **Karen:** | We man the BHC. We’re on standby for any orders the midwife will give us. For instance, we take charge of looking for patients who are missing in action. Patients usually approach us first if they have concerns that they can’t immediately open up to other providers. |
|  | **Leni:** | We do house-to-house visits to record the number of pregnant individuals and children. |
| ***IN:*** | *What is your main responsibility during a [local program on infectious diseases]?* | |
|  | **Leni:** | We take charge of reminding people to come to the center during their scheduled vaccinations. During mass check-up days, we also prepare the weighing scale needed for the check-up. We assist with weighing the child so the midwife can focus on vaccination. |
|  | **Josie:** | We also promote health programs to new settlers in the community. This is important since implementation of health programs differs from one area to another. |
|  | **Mari:** | We always remind them that this job is voluntary. We do regular house to house visits to know how many children and elderly there are. We also look at how many people need medication. |
|  | **Sally:** | We take care of the community by assisting them with health related concerns. We provide them advice that can help relieve their ailments. If we can’t provide a solution, we refer them to the midwife |
| ***IN:*** | *Are there specific responsibilities assigned to you?* | |
|  | **Leni:** | We regularly provide health advice. We give tips on how mothers can attend to the health related needs of their children. |
|  | **Karen:** | During vaccination days, we announce their schedule for vaccination. We also remind seniors to claim their medication at the center. We also advise them to go to the BHC to aid in their treatment and rehabilitation.  We also conduct regular house-to-house visits. This allows us to determine new occupants in the barangay. We introduce ourselves since this makes them more comfortable to approach us for any concerns. BHWs are also very involved in contact tracing since they know the ins and outs of the barangay. |
| ***IN:*** | *Someone mentioned that BHWs are involved in [a local program on environmental health], who wants to expound more about this?* | |
|  | **Josie:** | We visit houses to check the environment. We start by reminding them that they must clean their surroundings. We also help during clean-up drives so more work is done in a shorter amount of time. This order did not come from the RHU. The barangay tells us to involve ourselves in these initiatives.  BHWs don’t just involve themselves in health projects. We are immersed in various community-related activities. |
|  | **Sally:** | In some barangays like mine, BHWs take charge of distributing government aid. We hold the general profile of all residents. This makes us more familiar with who needs help the most. Creating a thorough profile is also important since this becomes the basis of budgetary allocations per barangay. However, this task can be disappointing. We advocate for those who really need aid. Some officials would still edit the list we provided.  It's disheartening. People would often come for us if aid is not provided to them. We can’t do anything about it really. We just execute orders from the top. |
|  | **Mari:** | It’s just disappointing really because we do a lot of work beyond our function. Despite this, we don’t receive much support from the barangay. |
| ***IN:*** | *Do you spearhead some activities for your constituents?* | |
|  | **Karen:** | I want to implement a lot of projects for my community. I can’t seem to make this happen. There is a lot of work to accomplish. |
| ***IN:*** | *How about in other barangays?* | |
|  | **Leni:** | We spearhead feeding activities. BHWs take charge of looking for logistics and encouraging people to come. The barangay captain just approves it. |
|  | **Karen:** | BHWs in my barangay conduct regular [local program on physical activity]. |
| ***IN:*** | *How would you compare tasks given by the RHU and the barangay?* | |
|  | **Josie:** | The gravity of tasks are almost the same. However, we prioritize orders given by the RHU since this is part of our mandate. |
| ***IN:*** | *What encouraged you to apply as a BHW?* | |
|  | **Mari:** | A friend encouraged me to apply. I appreciated the job since it gave me something to do during my free time. Eventually, I say myself in the profession for the long haul. I learned a lot of health related skills like bp taking and weight measurement. I also feel good since I’m able to help a lot of people. |
|  | **Sally:** | I didn’t expect to be assigned to the center. I just wanted a job in the public sector. I also wanted to take nursing back then. I had to stop school since we didn’t have enough funds to continue. Being a BHW however, helped me fulfill this dream.  Being a BHW also allows me to help my family. Besides this, BHW has significantly enhanced my communication skills. I am confident to interact with any kind of person nowadays. |
|  | **Karen:** | My mother was a BHW. I wanted to try what she did. Back then, BHWs were only in-charge of calling people. BHWs didn’t do paper work back them. This appealed to me.  The job description changed since then. However, I stayed as I find it fulfilling to help other people. |
|  | **Leni:** | I was inspired by my neighbor who worked as a nurse. I asked her to teach me how to take blood pressure readings. Since I had the basic skills, I applied as a BHW when the barangay called for applicants.  I’m in awe of our impact on sick people. I see people from our [local nutrition program] who are in better shape. This puts a smile on my face as I was able to restore their health. I also gain new friends from the senior citizens I get to interact with. |
|  | **Josie:** | My friend just encouraged me to apply. I still appreciated the job even when we were overwhelmed with responsibilities during the pandemic. It feels so good to be of great help to others. |
| ***IN:*** | *Do you receive benefits from the barangay?* | |
|  | **Josie:** | Our barangay provides us with an allowance. Sometimes, we are given materials for house-to-house initiatives. |
|  | **Leni:** | Supplies are regularly provided to us. There is a budget allotted for what we need. |
|  | **Mari:** | I worked without pay for one straight year. We were provided more allowance during the pandemic.  I just find it ironic that higher-ups tell us that there is a budget for health-related supplies. However, the barangay denies receiving any kind of budget. |
|  | **Karen:** | We have good barangay officials. We are free to do anything we want with our allowance. A separate budget is allotted for supplies. |
|  | **Sally:** | The barangay never responded to any of our requests. We have to spend out-of-pocket from time to time. This allows us to assess the needs of the BHC. |
| ***IN:*** | *What gives you the most fulfillment?* | |
|  | **Mari:** | We are recognized as heroes in our barangay. We are treated like family. |
|  | **Karen:** | They’re not afraid to approach us for any queries or concerns. They talk to us beyond their health concerns. |
|  | **Josie:** | When we distribute their medication, they tell us that they won’t achieve optimal health. This is so fulfilling to hear. You’re given the assurance that they’re good in health because of you. |
|  | **Sally:** | They treat us like family. They give us snacks and/or refreshments when we pass by. |
| ***IN:*** | *I see that you have a lot of tasks to execute. Does anyone guide you in accomplishing these?* | |
|  | **Karen:** | Orders mostly come from the NDP. They relay whatever projects are approved by the RHU. |
| ***IN:*** | *Do they give you guidelines and/or advice to help you execute these tasks?* | |
|  | **Leni:** | Yes, they do. Some patients have highly confidential diseases. The midwife and NDP provides us with relevant information about these diseases. They also give us advice on how to maintain confidentiality. They tell us whom to approach in case a patient has specific concern about their illness. |
|  | **Sally:** | We are given advice on how to handle pregnant teenagers. |
| ***IN:*** | *Do you solely rely on the advice of NDPs in executing your jobs or are personal strategies important?* | |
|  | **Leni:** | Coming up with our own strategies is very important. For example, we don’t approach pregnant teenagers in the same manner as they have varying interests and personalities. We give information to the patient depending on how prepared they are. We also consider internal family relations when giving advice. If needed, we ask them if they need help opening up about the pregnancy to their families. |
| ***IN:*** | *To clarify, do you have a significant role in helping patients open up to other people?* | |
|  | **Sally:** | Yes, we strategically talk to them. This helps them open up more to their families and to us. |
| ***IN:*** | *Do BHWs encounter particular challenges in the upland region?* | |
|  | **Angie:** | It’s hard to do house-to-house visits in far barangays. However, we take it upon ourselves to go to the patient’s house when they have difficulty leaving. We conduct health assessments in their homes instead of requiring them to commute to the health center. An example of this includes community to upland houses to get a patient’s BP. |
| ***IN:*** | *What is your most significant responsibility?* | |
|  | **Karen:** | Distributing medication is a big part of the job. Some patients advise us not to disclose their disease. We have to find a way to strategically distribute their medication without anyone knowing. |
| ***IN:*** | *What can possibly help to make convicting patients easier?* | |
|  | **Josie:** | It’s important to maintain our collaboration with the NDP and midwife. If ties are severed, we would lose direction. NDPs and midwives help a lot since they have extensive technical knowledge. |
| ***IN:*** | *Do you encounter any logistical difficulties?* | |
|  | **Leni:** | We don’t have enough weighing scales in the center. We have to find a creative way to measure the weight of pediatric patients. BHWs have raised this concern to the barangay. The barangay tells us that they can’t provide supplies immediately. Sometimes, we buy the weighing scales by ourselves just so we can weigh the kids. |
|  | **Mari:** | In our barangay, the kagawad gets frustrated when we ask for supplies. Officials tell us to find a creative solution to the shortage of supplies. If we can’t fix the weighing scale, we get money from the donation box. We also use money from our own allowance. The NDP and midwife also shells out money.  In most cases, this money is not returned to us, |
| ***IN:*** | *Does the barangay have a budget for material resources?* | |
|  | **Sally:** | Supposedly yes. However, we often have to use our own funds due to delays. |
| ***IN:*** | *You’ve frequently mentioned that being innovative is important in securing materials. Why so?* | |
|  | **Mari:** | Things won't be done if we aren’t innovative enough. Sadly, innovation mostly comes from necessity and the lack of support. We will be overwhelmed with a lot of work if we don’t fix things on time. BHWs are required to complete screenings regardless of the availability of materials. |
| ***IN:*** | *Do you like to improve or change something in the system?* | |
|  | **Karen:** | There needs to be a change in my barangay. We are always told that we don’t have a budget for health supplies. This leaves our BHC behind while other centers advance. I hope they genuinely allot funds for health programs. Otherwise, it seems like health is not the barangay’s priority. |
| ***IN:*** | *What is a good strategy to estimate the resources you need?* | |
|  | **Leni:** | It would help to have regular and documented consultations with BHWs. We don’t have consultations with the barangay. They are also unable to provide documentation of funding. |
|  | **Mari:** | BHWs in our barangay would get assigned to the daycare. Parents and BHWs would plan activities for the nearing graduation. Whenever the kagawad is approached about possible monetary assistance, he would always tell us that this is not possible.  They don’t have the initiative to help. I hope they learn how to immerse themselves in the community to identify what we need. |
|  | **Karen:** | I don’t encounter much problems in our BHC. I hope our center expands so we can give service to more patients. |
| ***IN:*** | *You mentioned that additional trainings will help. What trainings would you like to include?* | |
|  | **Josie:** | We hope to have more health related training. We still have a lot to learn when it comes to good and effective health practices. If this comes true, we hope to educate our communities more. We also hope there is training about maintaining cleanliness in the barangay. This topic is intricately related to health. |
|  | **Mari:** | I hope we have training on how to help mothers find a source of income. This is not health-related. However, it would go a long way if they learn how to support their families. I’m willing to teach these skills to my community if I’m given the chance to learn. |
|  | **Karen:** | We hope to receive more medication for all across the lifespan. A lot of people would ask us why seniors are the only one provided medication? We hope this service extends to everyone including us. We find it difficult when they argue with us but we really can’t give anything in return. |
|  | **Josie:** | There are available medicines in the RHU. However, people don’t have transportation going to the center. Irate patients would question our capacity as a center. They tell us that centers should be able to give medication for the sick. |
|  | **Mari:** | Our years in service have made us accustomed about giving advice to our patients. However, it’s very difficult to execute our jobs when we don’t have enough supplies. People tend to be less cooperative when there aren’t supplies in the center. |
|  | **Leni:** | OTCs are provided in the upland barangay I handle. However, these medicines are overpriced. The center is able to buy and provide less because of this pricing scheme.  We hope higher-ups also don’t change the documentation we provide. At one point, BHWs hosted a week-long activity. The kapitan requested us to declare that the activity occurred for a whole month. I had to ask him how to document this since it didn’t happen.  We are not given an allowance on some months. We were told that barangay officials lost our allowance letter.  We are so disappointed in the barangay. We are not given enough support. They can’t even provide us with snacks. |
|  | **Karen:** | On good days, we are given snacks during vaccination drives. We are provided a heavy carbohydrate snack on the first day. On the second day, only biscuits are provided. The snacks we receive get smaller on subsequent days. We often don’t receive anything on the 4^th^ or 5^th^ day. |
| ***IN:*** | *Would you like to change anything in terms of documentation or assessment?* | |
|  | **All:** | No, I am able to handle it. |
|  | **Mari:** | I hope our workload gets reduced. Responsibilities that can be allotted to the kagawad must be allotted to them. We already voiced this out to the barangay. Back then, we were also assigned to distribute [a government document]. The barangay told us that we should do this since we know most people in the barangay.  The kapitan would permit us to delegate this to the tanod. Unfortunately, we are also asked to take on some tasks from public school teachers. |
| ***IN:*** | *How about your health related tasks?* | |
|  | **Karen:** | We are willing to do all health-related tasks assigned to us. This is part of our mandate. |
|  | **Sally:** | It’s not hard for me to accomplish documentation related tasks. It is quite extensive. However, I’m able to accomplish such as long as I pencil it in my schedule. |
| ***IN:*** | *Does technology help in managing workload?* | |
|  | **Sally:** | Having a cellphone helps. Reaching out to our constituents is more efficient. |
|  | **Josie:** | I created a group chat with my patients. This helps me make a uniform and immediate announcement of all health related programs in my barangay. |
|  | **Mari:** | I use our gc to announce the names of pediatric patients who need to go to the center. I’m also able to monitor who seenzones me. This helps me plot whom to do face-to-face visits with. |
|  | **Karen:** | I would run out of patience from time to time. Some parents are really negligent. We tell them that it seems wrong to have money for vices when they keep on telling us that they don’t have enough cash to bring their kids to the BHC. The parent changed after I confronted her. As an extra measure, we ask a service to get them if one is available. |
| ***IN:*** | *Does having a phone help you reach out to the barangay’s shuttle service?* | |
|  | **Mari:** | Yes, we immediately know if someone is available to fetch patients or not. We relay this immediately to parents so they can prepare in advance. Having a service is helpful for patients who tend to change their minds a lot. It’s easier to fetch these patients when these patients suddenly feel that it’s time for a check-up. |
|  | **Sally:** | Cellphones are helpful in upland barangays since houses are far from each other. When I call patients in advance, I’m able to prepare the supplies I need to bring to their house. |
| ***IN:*** | *You mentioned that having a phone helps you do your tasks. Is a load allowance provided?* | |
|  | **Josie:** | No. Load comes from our own pocket. BHWS are prohibited from connecting in Our BHC’s Wi-Fi. The NDP has requested a Wi-Fi budget. This has not been utilized in our barangay. |
|  | **Karen:** | BHWs are allowed to connect in our barangay. However, the signal is bad. This is slightly better than not being allowed to connect. |
| ***IN:*** | *Let’s proceed to discussing your experiences with UTD. How often do you use UTD outside the journal club?* | |
|  | **Karen:** | I experienced using UTD for work. A high blood patient went to the center. I didn’t know what to do at first. I used UTD to check for possible remedies since our midwife was off duty. Here, I discovered that it’s best to bring the patient to the RHU. UTD helped me understand that he must rest for a few minutes before being transported. I helped solve this emergency when others were off duty. |
|  | **Sally:** | My child had a sore throat. I used UTD to search for any dietary restrictions. I also learned that he needs medication through UTD. However, I haven’t used this application for my job. |
|  | **Mari:** | I’m unable to access UTD. We are not allowed to connect to the BHC’s Wi-Fi. |
| ***IN:*** | *What device did you use to access UTD?* | |
|  | **All:** | Cellphone. |
| ***IN:*** | *What other factors encourage you to use UTD?* | |
|  | **Karen:** | It’s helpful when we do advance reading for journal clubs. It helps us have a basic understanding of the disease before the lecture. |
| ***IN:*** | *What discourages UTD use?* | |
|  | **Josie:** | I don’t use it that often because of internet connection difficulties. |
|  | **Leni:** | I prefer to use Facebook. |
| ***IN:*** | *Based on your responses, UTD acceptance is low among BHWs?* | |
|  | **Mari:** | Yes it is. There are so many tasks to accomplish. We apply what we already know to help patients in our care. |
| ***IN:*** | *How do you find the application’s content and layout?* | |
|  | **Karen:** | It’s hard to read the content. We can translate it to Filipino. However, the translation is either deep and/or inaccurate. |
|  | **Josie:** | The content is too long. Direct to the point content allows us to immediately apply learnings to the patient. It’s difficult to provide overly detailed information. It would also help if images are included in the readings. |
|  | **Sally:** | Integrating more audiovisual components is helpful. Not all are good in English. Videos help us visualize what to do. |
| ***IN:*** | *How do you find journal clubs?* | |
|  | **Karen:** | I hope lectures are more simplified. At times, we feel like the content of seminars is different from what we read in UTD. Having side to side comparisons would help so we can identify similarities and differences between UTD and the lecture. |
|  | **Josie:** | It’s hard to absorb the lecture when the internet connection is unstable. |
| ***IN:*** | *Would it help if more real-life applications are provided?* | |
|  | **Leni:** | Yes, it will. Actual demos would also be helpful. This would help us identify the exact role of BHWs in managing different diseases. |
| ***IN:*** | *Would you recommend UTD to other PCPs.* | |
|  | **Mari:** | Yes, if needed. My sister has a uterine disease. UTD can help her identify possible remedies. It might also give her the urgency to consult a doctor.  She’s a midwife here in Samal. I’m quite surprised that she hasn’t heard about UTD before. |
|  | **Karen:** | UTD will help especially when there are staff shortages. UTD can help us manage patients even when the midwife is not on duty. This also makes us less reliant on the RHU. This helps us assist patients especially in emergency situations. |
|  | **Leni:** | Yes, I will. Everyone in the health sector should have familiarity with the application. |
| ***IN:*** | *Some have mentioned that BHWs prefer to get information from Facebook. Why so?* | |
|  | **Sally:** | A load allowance does not guarantee UTD use. Others will definitely prefer Facebook. |
|  | **Leni:** | I can convince others by telling them that UTD will help us improve. We can definitely get information from Facebook. However, UTD may help us improve more since it’s a credible reference.  Although it’s quite difficult to convince others. A lot of them are hooked on Youtube. |
|  | **Josie:** | Everybody prefers vlogs, google, or Facebook. They can immediately retrieve the information they need. |
| ***IN:*** | *Will video materials help?* | |
|  | **Karen:** | Yes, it would. You can immediately identify the application of concepts. Reading a lot of concepts can be overwhelming. |
|  | **Leni:** | I will emphasize that UTD is credible. Hence, it should be our priority. However, information should be presented in a way that appeals to the audience. |

**--[End Transcript (1:21:10)]—**
