# Supplementary material for: Resilience, ingenuity, and identity: A multi-level analysis of the Filipino community health worker experience in rural and remote municipalities in the Philippines
Source: PLOS Glob Public Health. 2025 Aug 18;5(8):e0004965. doi: 10.1371/journal.pgph.0004965 (PMC12360505; doi:10.1371/journal.pgph.0004965)
Supplement: Attachment — Submitted filename: 2024-12-29 PLOS Global Public Health Response to Comments.pdf [file pgph.0004965.s006.pdf]

PEER REVIEWER COMMENTS AND FEEDBACK
Response Form
Due July 20, 2025
PLOS Global Public Health Decision: Revision Required

**Source**	**Comments**	**Response**
Review 4	1. The manuscript is well organized, specially after peer review process. But still it needs to be re stated the research gap, it is weak to my perspective. Also consider to explain why you use this method to address this gap.	Thank you for this feedback! We believe that this is crucial in helping us improve the quality of our manuscript. You may refer to our response for the requested revision below:<br>1. A research gap has been integrated as a separate section in the methodology of our manuscript (page 5). This section highlights the need for the study and a brief justification as to our chosen method for data collection.
