# Supplementary material for: Resilience, ingenuity, and identity: A multi-level analysis of the Filipino community health worker experience in rural and remote municipalities in the Philippines
Source: PLOS Glob Public Health. 2025 Aug 18;5(8):e0004965. doi: 10.1371/journal.pgph.0004965 (PMC12360505; doi:10.1371/journal.pgph.0004965)
Supplement: Attachment — Submitted filename: 2024-12-29 PLOS Global Public Health Response to Comments.docx [file pgph.0004965.s005.docx]

**PEER REVIEWER COMMENTS AND FEEDBACK**

Response Form

Due November 30, 2024

PLOS Global Public Health Decision: Revision Required

| **Source** | **Comments** | **Response** |
| --- | --- | --- |
| Journal Comments | 1. Please clarify all sources of funding (financial or material support) for your study. List the grants (with grant number) or organizations (with URL) that supported your study, including funding received from your institution. 2. State the initials, alongside each funding source, of each author to receive each grant. 3. State what role the funders took in the study. If the funders had no role in your study, please state: “The funders had no role in study design, data collection and analysis, decision to publish, or preparation of the manuscript.” 4. If any authors received a salary from any of your funders, please state which authors and which funders. 5. In the online submission form, you indicated that "Data can be made available by the authors upon reasonable request.". All PLOS journals now require all data underlying the findings described in their manuscript to be freely available to other researchers, either  1. In a public repository; 2. Within the manuscript itself, or; 3. Uploaded as supplementary information. 6. Please provide separate figure files in .tif or .eps format. | Thank you for this feedback. The PPCS team deeply values all feedback from the Journal. We believe that these are essential to ensuring the clarity of our research. Our team of authors has worked towards addressing the comments. Please refer to the itemized list of responses for each feedback:   1. The source of funding has been stated in **the 3^rd^ paragraph of Page 26**. The URL included will direct the editors to the public web page of the funder. However, no grant number was provided by NAST. Hence, none is indicated in the manuscript. 2. NAST awarded the grant to the Philippine Primary Care Studies. The funding was used to sustain different aspects of the CHW Study. Our team clarifies this detail in the **3^rd^ paragraph of Page 26**. 3. The funder (NAST) took no role in crafting the study design, data collection, and analysis. We have integrated the suggested statement in the **3^rd^ paragraph of Page 26**. 4. We included the authors who received a salary for this study in the **4^th^ paragraph of Page 26**. 5. We value this feedback. The authors have uploaded this as supplementary information. We have anonymized and de-identified the necessary data. 6. All figures have been re-uploaded in .tif format.   Once again, our team expresses our gratitude for taking the time to review our study. We hope these revisions shed more light on the unique experience of Community Health Workers in the Philippine setting. |
| Reviewer 1 | 1. This is a well-written manuscript providing evidence of known successes and challenges regarding the performance of CHWs. I do not have major substantive comments for the others, just a couple of suggestions for consideration to improve the manuscript.  The first is to find more updated numbers for the CHWs rather than the 2015 that is referred to in the background if available.  The second is to put some of the context content that is in the policy recommendation earlier in the documents so that the section focuses more on what the authors are recommending. Currently, these are buried in the explanation. | Thank you for taking the time to review our study on the experiences of Community Health Workers. Your feedback was very essential in allowing us to solidify the unique challenges faced by CHWs that must be addressed.  Our team has found a reliable updated count of all CHWs on active duty. The number of active CHWs rose from 235,653 in 2015 to 241,282. This change is reflected in ***the 1^st^ paragraph of page 3***. Both the in-text citation and the bibliography have also been updated.  Discussion on the context content has been integrated into the ***Background section*** of the manuscript. We believe that its integration into the ***2^nd^ paragraph of page 4 provides*** an in-depth sequence of the laws that were previously implemented to provide support for CHWs on the policy level. This paragraph further discusses how despite the implementation of these laws, CHWs remain in a compromised position due to a lack of awareness among CHWs themselves and differing priorities among local government units.  These changes have allowed us to shed more light on our policy recommendations. We provided an expanded discussion on enhancing financial stability among CHWs through the formation of organized credentialing systems and the implementation of regular collaborative meetings. Accordingly, we also expanded our discussion on increasing training opportunities for CHWs. Our suggestion to create collaborative learning opportunities that integrate theoretical and practical training is discussed in more detail. These suggestions are integrated from the ***second paragraph of page 23 to the 1^st^ paragraph of page 24***.  Once again, we thank the reviewer for their thoughtful insights on our manuscript. Your feedback has genuinely allowed us to discuss the context of CHW experiences within the Philippine setting while providing more comprehensive insight into policy. |
| Reviewer 2 | This is an interesting paper on an important topic that is currently receiving a lot of international attention again. Key comments for revisions:  1. The authors explain that this study is based on the secondary analysis of data collected for a parent program piloted a study on a clinical decision support tool for CHWs. However, it does not become clear in the methods how the original data were read and analyzed to answer a different set of questions and objectives for this paper. Both the abstract and the methods section of the main paper need to elaborate on this.  2. As a reader from another region of the world I would need a brief overview of the structure and levels of the Philippine health system to be able to understand how CHWs are located within it.  3. There appears to be contradictory information about the scope and role of CHWs: the introduction mentions literature that says that they can only work under direct supervision from nurses and midwives, yet the main text paints a much more diverse and possibly fragmented picture. This would need clarification, including an explanation of how the UpToDate tool changes CHWs' scopes and roles.  4. It is unclear how the role descriptions in Table 3 were generated, i.e. whether they are self-reported by CHWs. Overall the evidence base for the findings presented remains quite opaque to the reader.  5. The discussion currently continues to present results. It does not constitute a discussion of the presented findings.  6. A minor issue: "Diskarte" is a term that is unfamiliar to me and would need to be explained. | We thank the reviewer for taking the time to provide key insights on our manuscript. Our team believes that this feedback allow us to shed more clarity on the unique experiences of CHWs within the Philippine setting. Our team of authors worked towards addressing your comments. Please refer to the itemized list of responses for each feedback:   1. In the methods section of the abstract, we clarified that FGDs for the parent study explored the experiences of CHWs with a clinical decision support tool. However, the group interviews also emphasized the roles of CHWs and the factors that influence their performance in great detail. Hence, we deem this subset of the FGD data useful for analyzing the barriers and enablers for the role performance of CHWs. We further elaborate on this in the methods section of the manuscript. ***Under section 2.4***, we emphasized the interview tool used for this study including questions on the compatibility of using UpToDate (clinical decision support tool) with existing workflows and the experiences of CHWs within their local health settings. A separate study explored CHW insights on UpToDate use. Analysis for this study centered on questions that looked into the CHWs’ daily responsibilities, available resources, and factors for role performance. We further emphasized that a mixed inductive/deductive approach was used to analyze this subset of the FGD data. 2. We addressed this feedback by providing more context on how the present structure of the Philippine Health system came to be. In the **3^rd^ paragraph of page 3**, we emphasize how the passage of RA 7160 shifted the decision-making responsibility from the national government to the local level. The Philippine Healthcare system follows the three levels of care system. CHWs are primarily located at the primary level of care as their duties primarily focus on health promotion and disease prevention. Despite this standardized setup, the implementation of RA 7160 has greatly contributed to the variability of roles and responsibilities within each locality. An in-depth discussion of these potential variabilities can be found in the **1^st^ paragraph of page 4**. 3. We confirm that CHWs work under the supervision of nurses and midwives. Their use of UpToDate allows CHWs to gain more understanding of the health conditions of their patients and potential health teachings to resolve their patients’ concerns. However, it is not used to diagnose patients or treat them through medical interventions as these remain the responsibilities of licensed care professionals. While some CHWs have suggested potential diagnoses to patients, they always refer their patients to a physician for a medical diagnosis. We further emphasize that CHWs stick to the provision of light advice such as non-pharmacological interventions to avoid patient harm. These insights are reflected in the following sections of the manuscript:   ***3^rd^ and 4^th^ paragraph of page 12***  ‘The role of CHWs as direct service providers, however, is relatively narrow. Their duties are primarily limited to blood pressure monitoring and administering first aid if other providers are unavailable. While a few CHWs recalled instances when they suggested diagnoses to patients, most exercised caution due to their fear of potentially providing inaccurate interpretations of medical conditions. This cautious approach led them to refer patients to the supervising midwives or the primary care physicians stationed at the central health unit. Gina, a long-tenure CHW, described:  *"When mothers message me on Facebook about their child's symptoms, I consult UpToDate [a digital clinical decision-support tool] to find what can be done to improve the child’s condition. However, I stick to providing light advice. I'm afraid to interpret complex health information, as I could easily make mistakes and potentially harm the patient."*  ***5^th^ paragraph of page 18***  “Experiences of CHWs who frequently used UpToDate also reported that clinical support tools augmented self-efficacy in the field. The decision to perform medical interventions remains the responsibility of physicians. However, tools such as UTD enhance their knowledge base on various diseases while allowing midwives to learn about non-pharmacological measures they can suggest to help the patient. Where other CHWs may have sought directions from a midwife, frequent UpToDate users described that they were able to rely on their decision-making with the help of these tools”   4. We clarified that the roles presented in Table 2 were identified through an analysis of participant accounts. This clarification is reflected in the **2^nd^ paragraph of page 10**. 5. We appreciate the second reviewer’s feedback on the structure of the manuscript’s discussion section. This is the first time a Philippine study expands on the barriers and enablers of CHW role performance within their local health settings. We believe this structure is appropriate as it allows us to emphasize what participants have directly said while providing a thorough interpretation of their insights. 6. We further expound on the concept of discharge in the ***3^rd^ paragraph of page 14***. Beyond explaining how it manifests in Filipino culture “When confronted with resource deficits, CHWs employed self-devised strategies or their *diskarte* to navigate emergent challenges. *Diskarte* can be defined as an individual’s ability to overcome situational limitations to ensure survival. However, unlike resilience or resourcefulness, its use denotes not only a lack of resources but also unequal social positions” We expounded how it can manifest in healthcare settings “This usually involves creating strategies to improve a situation despite the limitedness of support and resources. In healthcare settings, its repeated use is fueled by the poor implementation of policies to secure the needs of the workforce.”   Once again, the team expresses its gratitude for the reviewer’s feedback on our manuscript. We hope these revisions pave the way for a better of the unique experiences of CHWs within the Philippine setting. |
| Reviewer 3 | This is a very nice study reporting the insights and opinions of community health workers in the Philippines. I find the paper incredibly well done and very interesting; I was fascinated with the analysis and learned a number of new ideas.  In particular, I appreciate the authors introducing a new audience to the concept of “discharge.” This term points towards something I have seen in community health programs across multiple continents, even if the word itself is from a local language.  Similarly, I appreciated learning about the term “marites.” I was fascinated that it held both positive and negative connotations and that the community health worker who shared this term wore it as a (perhaps embarrassing?) badge of honor. This duality mirrors the contradiction that many community health workers are forced to live - that their jobs bring both positivity and negativity into their lives. But this ability to be positioned as a “marite” is likely a product of their personalities and talents in being able to relate with people interpersonally. It likely also comes from an inborn curiosity, and willingness to serve. As has previously been said, “Community health workers are not trained, they are born.” Only the “marite” can get that deep into the community, and this position brings back new functionality to the health system.  Otherwise, the paper reported on dynamics that have been well described in countless other papers, though the authors do a good job of explaining how all this works in the Filipino context.  The only suggestions that I would recommend, with the aim of further improving the paper, are as such:   - I recommend that the authors consider a new title. When I first read the title, I found myself not interested in reading further. I doubted whether or not we needed another paper looking at barriers and enablers in community health. The term “an unpaid labor of love“ also does not fully capture what they are trying to report, because it sounds like because it is “a labor of love”, it can (should?) remain underpaid. Maybe they can find a better title by leaning into one of the insights I mention aboveBasically, what I think this paper adds is a very good overview of the Filipino context, and uniquely local conceptualizations of how the Filipino woman is situated in health work (marite) and how they bring to their jobs incredible ingenuity (diskarte). - Similarly, the abstract does not capture the analytic depth or complexity of the study. For example, the authors claim that this study is necessary because “barriers and enablers… remain underrepresented in existing literature.“ This is simply not true, as this topic has been extensively studied. What this study actually is doing is pulling from the Filipino context unique insights that are relevant for community health workers everywhere, and their multinational struggle for professionalization. I recommend the authors look at multiple other articles reporting on "barriers and enablers," and to find language not common in those papers. For example, when the authors mention in the abstract about the need for "legal safeguards," it sounds cliched; what they are describing is the incredible experience of decades of existing laws being poorly implemented. This inability to make existing community health laws have teeth invaluable information for the current struggle to support community health everywhere, but a reader wouldn't know that just looking at the abstract. I also would avoid the term "unsustainable;" what I hear is that the position of a community health worker in the Philippines HAS been sustained for decades, but it is just poorly supported and poorly positioned. I could go on line-by-line, but this is true for most of the abstract; basically, the paper is much more complex, nuanced and original, and I wish the abstract mirrored that better. - Finally, while I think that the authors are very well read in community health, there is a wide variety of activity that they should know about. - I refer them to the National C3 Council effort, to further define CHW roles, which mirrors and somewhat built upon what they report. https://www.c3council.org - I also refer them to the work of the Community Health Impact Coalition (CHIC), which is a multinational coalition of non-governmental organizations working to make professionalized community health workers the norm worldwide. Many of the publications produced by this group at CHIC may be helpful for supporting this current analysis, or future analyses. | We thank the reviewer for their encouraging comments on our manuscript. Indeed, the concepts of “diskarte” and “marites” were crucial data points of the study that highlighted the unique experiences of CHWs within the Philippine setting. Accordingly, the team has worked on addressing the specific comments. Below are our responses to the feedback provided:   1. Our team has revised the title to “Resilience, Ingenuity, and Identity: A Multi-Level Analysis of the Filipino Community Health Workers Experience in Rural and Remote Municipalities in the Philippines”. We believe that this reflects the unique contributions of our manuscript to the literature on community health workers. The revised title emphasizes the resilience, ingenuity, and unique identities that they embody in everyday practice. The context of this revised title is strongly nuanced and comprehensively discussed in the results and discussion of our manuscript. 2. The team agrees with this feedback. We acknowledge that professionalization is a multinational struggle among CHWs. In **the background of the abstract**, our team clarifies that this manuscript focuses on providing more insight into barriers and enablers present in the Philippines. CHWs navigate these experiences by drawing on cultural knowledge and experiences rooted in the Philippine healthcare setting. We further acknowledge that a stronger implementation of laws already in place is what’s necessary to improve the current situation of CHWs. Beyond passing laws, LGUs must be genuinely accountable for ensuring its implementation. We also acknowledge that individual efforts will only bear fruit if supplemented with a solid support system on the policy level. To this end, the **conclusion** of our abstract emphasized that support from national and local health leaders is crucial to ensuring that the rights of CHWs are upheld.   Thank you for your referral to CHW literature. These resources have genuinely helped us gain a more comprehensive understanding of the experiences of CHWs in different settings. We share the belief that there is great value in bringing their experiences to light. It provides stronger emphasis on the measures that need to be done to strengthen the protection and safeguards that CHWs deserve. |
